# Critical Evaluation of Key Elements in Manufacturing Procedures and Testing Methodologies for Ocular Anti-Infective Thin Film Inserts

**DOI:** 10.3390/ph19081222

**Published:** 2026-08-04

**Authors:** Alfredo Desiato, Affiong Iyire, Raquel Gil-Cazorla

**Affiliations:** 1 Optometry and Vision Science Research Group (OVSRG), School of Optometry, Aston University, Birmingham B4 7ET, UK; a.desiato1@aston.ac.uk; 2 Aston Pharmacy School, College of Medicine, Pharmacy and Biosciences, Aston University, Birmingham B4 7ET, UK; a.iyire@aston.ac.uk

**Keywords:** ocular drug delivery systems, antimicrobial ophthalmic inserts, polymeric thin films, solvent-casting manufacturing, controlled drug release, ophthalmic antimicrobials, in vitro evaluation, pharmaceutical formulation development, pharmaceutical formulation testing

## Abstract

Eye drops remain the principal topical treatment for ocular infections, yet rapid precorneal clearance, variable dose delivery and limited tissue penetration can restrict local drug availability and necessitate frequent administration. Conjunctival inserts have long been investigated as a means of extending ocular residence and thin film inserts offer a more adaptable solid dosage form that may provide a defined drug-containing unit, prolonged local exposure and hydration-dependent dissolution or transformation within the conjunctival sac. Clinical translation remains limited by substantial variability in formulation design, manufacturing control and performance testing. This review critically evaluates the manufacture and characterisation of ocular anti-infective thin film inserts, with the aim of identifying the principal factors that determine reproducibility, interpretability and progression beyond formulation feasibility. Film architecture, polymer selection and drug-loading strategy are considered in relation to the physicochemical characteristics of the active pharmaceutical ingredient and the intended behaviour of the finished insert. Solvent casting remains the most extensively investigated manufacturing approach, while extrusion, electrospinning and additive manufacturing broaden the available processing options. Across these methods, incomplete specification of material and process variables frequently restricts comparison and reproducibility. Testing procedures are similarly heterogeneous and often assess individual attributes without establishing how the finished insert performs under conditions relevant to conjunctival administration. Particular limitations concern dosage-unit uniformity, hydration and matrix transformation, drug-release models and the interpretation of antimicrobial activity. Progression towards clinically relevant products will require indication-led development in which manufacturing control, pharmaceutical quality, ocular compatibility and biorelevant performance evaluation are considered as connected elements. This approach may provide a stronger basis for determining whether the potential advantages of ocular anti-infective thin film inserts can be translated into reproducible and clinically useful dosage forms.

## 1. Introduction

Eye drops remain the predominant topical dosage form used for the treatment of ocular infections [1]. The amount of active pharmaceutical ingredient (API) reaching the intended site depends on both the persistence of the formulation on the ocular surface and the ability of the drug to penetrate the relevant tissues [2,3,4]. The limited volume that the eye can accommodate, together with tear-film dilution, blinking, tear turnover and nasolacrimal drainage, results in rapid precorneal loss following instillation [2,3]. Where access to the cornea or deeper anterior tissues is required, drug availability is further restricted by the corneal epithelium, whose intercellular junctions and lipophilic characteristics limit the passage of many hydrophilic and higher-molecular-weight compounds [4,5], and a proportion of the dose is also removed through the nasolacrimal pathway and may undergo systemic absorption, rather than contributing to the intended local effect [6].

The volume delivered through eye-drop administration is inherently variable, as drop size can be influenced by the physicochemical properties of the formulation, the design of the bottle and dispensing tip, container orientation and instillation technique [7,8]. This variability is particularly relevant to anti-infective treatment, for which adequate local drug concentrations should be maintained in relation to the susceptibility of the causative microorganism [9]. Frequent administration is commonly required, with particularly intensive regimens used during the initial management of more severe ocular infections [9,10], which may involve several topical instillations at short or differing intervals, resulting in a substantial medication burden for patients and caregivers [10]. Frequent and complex dosing can also increase the practical demands of treatment and may contribute to incomplete adherence or administration errors [1,11].

Several non-solid formulation strategies have been investigated to increase precorneal drug retention and facilitate drug transfer across the ocular tissues [3,12]. Polymers have been incorporated into ophthalmic formulations to modify viscosity, mucoadhesion, gelation, drug solubility and release, thereby influencing residence and ocular drug availability [13,14]. Suspensions and emulsions can facilitate the topical administration of drugs with limited aqueous solubility, while ointments may extend ocular contact through their semi-solid consistency [1,15]. The transient visual disturbance and reduced convenience associated with ointment use may, however, limit their acceptability for daytime administration [15]. Viscosity-enhancing and mucoadhesive polymers can delay drainage or promote interaction with the ocular-surface mucin layer [14,16]. In situ gelling formulations are administered as liquids and undergo gelation or a marked increase in viscosity in response to ocular temperature, pH or tear-fluid ions, combining the convenience of drop administration with extended precorneal retention [17,18]. Permeation-enhancing excipients have also been investigated to increase epithelial drug passage, although any improvement in penetration should be considered alongside the potential for ocular-surface barrier disruption and local cytotoxicity [19,20].

Micro- and nanocarrier systems, including polymeric particles, liposomes and nanomicelles, can accommodate drugs with different physicochemical characteristics and modify their apparent solubility, release, precorneal retention and penetration into ocular tissues through variations in carrier composition, size and surface properties [21,22,23,24]. Their effects on drug solubilisation, release, retention and tissue penetration should be considered as distinct formulation outcomes, as improvement in one attribute does not necessarily result in a comparable improvement in the others [3,12,24]. When administered as liquid dispersions, carrier-based formulations remain dependent on successful instillation and continue to be affected, to differing extents, by variability in the administered dose, tear dilution, blinking and nasolacrimal drainage [3,14,24]. The transition from conventional eye drops towards prolonged or controlled delivery should therefore be assessed according to the combined effects on dose administration, precorneal residence, drug release, tissue penetration and local drug availability, rather than on release duration alone [1,5].

Preformed solid and device-based systems have been investigated as a means of reducing dependence on repeated instillation by retaining a drug-containing structure at the ocular surface, within the lacrimal drainage pathway, or closer to the intended ocular tissue [5,25]. Among these, drug-eluting contact lenses can maintain direct contact with the cornea during wear while simultaneously correcting ametropia, and several approaches (including molecular imprinting and the incorporation of drug-loaded particles or films) have been explored to modify drug loading and release, also in view of the inherently limited volume of the lens matrix [26,27]. Their suitability for drug delivery, therefore, remains dependent on preserving optical performance and transparency, as well as the physiological and fitting characteristics required for lens wear, while contact-lens-related ocular-surface risks must also be considered [26,27,28]. The recent approval of a drug-eluting contact lens for allergic conjunctivitis demonstrates that this approach can reach clinical implementation, although the device has been discontinued and was contraindicated in the presence of corneal infection, therefore not providing a suitable platform for anti-infective delivery [29].

On the other hand, intracanalicular inserts, peri-/intra-ocular implants and microneedle-based systems similarly seek to extend drug exposure to ocular tissues, while reducing reliance on patient-administered dosing or facilitating passage across ocular barriers [25,30,31]. Depending on their design and anatomical location, these approaches may, however, require professional placement and/or removal by means of invasive or surgical implantation procedures [25,30,31,32]. Microneedles containing anti-infective agents, including dissolving antibiotic-loaded systems, remain supported by experimental and preclinical evidence and require precise administration to ocular tissues [32,33], while still raising considerations related to invasiveness, visual function, handling, persistence, and patient comfort [5,25].

Conjunctival inserts represent another group of preformed solid ocular systems, designed to prolong residence within the conjunctival sac without requiring an invasive administration procedure or surgical implantation [25,34]. Their characteristics have ranged from insoluble membrane-controlled reservoirs requiring retrieval after use (e.g., Ocusert^®^) to soluble drug-containing matrices intended to hydrate and dissolve in the tear fluid (e.g., SODI and NODS), and rod-shaped or temporary inserts developed for specific indications (e.g., Lacrisert^®^ and Mydriasert^®^) [35,36,37,38,39]. By providing a discrete unit and extending contact with the ocular surface, these systems can modify local drug/polymer exposure relative to eye drops, although their performance remains dependent on their architecture, retention, and behaviour after administration—including their influence on vision [34,37,40,41].

Although conjunctival placement can avoid an invasive procedure for administration, it introduces practical and tolerability considerations associated with retaining a preformed device within the conjunctival sac. Difficulties with insertion or positioning, foreign-body sensation, increased lacrimation, movement or expulsion, transient visual disturbance, and discomfort may limit acceptability or shorten the intended residence [25,34,42,43]. Soluble systems may avoid planned retrieval but require sufficiently predictable matrix hydration and dissolution, whereas insoluble or temporary inserts remain dependent on reliable retention and removal, where applicable [25,44]. These trade-offs may partly explain why conjunctival inserts have remained confined to a limited range of indications, rather than becoming broadly adopted alternatives to eye drops [25,45].

The transition from conventional eye drops to controlled-release ocular delivery involves, however, more than extending the duration of drug release, and controlled-release systems have to provide, therefore, reliable placement and retention, predictable release of an active pharmaceutical ingredient, and sufficient local availability without compromising vision, comfort or ocular-surface integrity [5,25,46]. Within this landscape, thin film inserts have been investigated as preformed polymeric dosage forms intended principally for placement within the conjunctival fornix, where delivery of the API as a discrete unit may support prolonged ocular-surface residence and more controlled release without requiring tissue penetration or surgical implantation [1,46,47]. Their relatively thin and flexible configuration may reduce the bulk associated with some inserts’ design, while variation in the polymeric matrix can allow the dosage form to remain intact or progressively dissolve, erode or undergo gel transformation following contact with tear fluid [42,46,48]. These characteristics may therefore combine sustained local delivery with avoidance of planned retrieval, although their relevance depends on the behaviour of the finished insert after administration rather than on its film configuration alone [46,48,49].

However, the suitability of thin films for ocular use cannot be inferred from their release profile alone, as their dimensions, mechanical behaviour and surface characteristics may influence insertion, positioning, retention and comfort, while hydration and matrix transformation can modify both residence and drug release [42,46,49]. Excessive swelling, fragmentation, incomplete dissolution or prolonged persistence may consequently compromise local tolerability or produce an exposure profile that differs from that anticipated through conventional in vitro testing [46,48,49]. Similarly, preparation as an individual drug-containing unit does not itself establish dose uniformity, while sustained release cannot be interpreted independently of ocular residence, tissue penetration, retained antimicrobial activity and biological compatibility [1,49,50]. Accordingly, the potential advantages of thin film inserts require linked evaluation of formulation, manufacture, pharmaceutical quality, ocular behaviour, and delivery of active drug to the intended site [1,46,48].

Published work on anti-infective ocular thin films remains centred predominantly on formulation development and preclinical evaluation, with considerable variation in composition, architecture, drug-loading approaches and manufacturing procedures [1,48,49]. Progression beyond proof-of-concept development therefore requires more than prolonged drug release, as the insert must also be manufactured reproducibly, administered and retained acceptably, and provide active antimicrobial exposure without compromising ocular compatibility under conditions relevant to clinical use [1,25,46,48]. However, these interdependent elements have often been evaluated separately using heterogeneous or incompletely reported methods, limiting the extent to which favourable findings for individual attributes can support scale-up, regulatory assessment or clinical translation [1,47,49].

## 2. Film Manufacturing Processes

### 2.1. Manufacturing Approaches for Ocular Anti-Infective Inserts

Ocular anti-infective inserts have been manufactured predominantly by solvent casting, while hot-melt extrusion, electrospinning and additive manufacturing have been investigated less extensively as alternative approaches [51,52,53,54]. Their principal characteristics, potential advantages and critical limitations are summarised in Table 1. These strategies differ in their material requirements, processing conditions and resulting dosage-form structures, and their suitability should, therefore, be assessed in relation to the physicochemical and thermal properties of the API, polymer processability, intended architecture and finished-product specifications [47,52,55].

Solvent casting remains the most widely reported approach and has been used to prepare monolithic, carrier-containing and multilayered systems [48,57,61]. Its accessibility and compatibility with aqueous or mixed-solvent formulations favour laboratory-scale development, although the resulting films remain sensitive to formulation rheology, mixing and deaeration, deposited volume or wet-film thickness, substrate characteristics and drying conditions [48,57]. Manual casting into dishes or moulds may also provide limited control of thickness and spatial uniformity and may not translate directly to continuous or larger-scale manufacture [48,62].

Hot-melt extrusion replaces preparation and drying of a solvent-containing formulation with thermomechanical processing of the API and excipients, thereby shifting the principal requirements towards material softening, melt flow and stability under heat and shear [52,63]. The process can produce continuous strips or sheets for subsequent subdivision, or filaments for fused-deposition-modelling manufacture, without introducing a solvent-removal stage [52,54,63]. Avoidance of processing solvents does not remove formulation constraints, as suitability remains dependent on plasticiser concentration, feed behaviour, screw configuration, barrel-temperature profile, residence time and the thermal and mechanical stability of the API and excipients [52,63]. The comparatively limited ocular anti-infective evidence therefore supports technical feasibility more clearly than broad applicability across APIs and polymer systems [52,54].

Electrospinning produces non-woven fibrous matrices rather than continuous films and should therefore be considered a structurally distinct insert-manufacturing approach rather than a direct procedural substitute for solvent casting [53,64]. Fibre composition, diameter, porosity and sequential deposition can be modified to influence hydration, structural persistence and drug release, while the resulting characteristics remain sensitive to interdependent formulation, electrical and environmental variables [53,64,66]. The high surface area and limited thickness of some fibrous mats may facilitate hydration but can also increase the importance of handling, structural stabilisation and allocation of the deposited material into dimensionally and compositionally defined units [53,65].

Additive manufacturing encompasses several non-equivalent routes rather than a single film-production method, with reported approaches including fused-deposition modelling, direct powder extrusion and extrusion-based deposition of semi-solid or carrier-containing formulations [54,67,69,70]. Digital control can define external geometry and selected internal design parameters, although the achievable structure remains conditional on platform resolution and the processing behaviour of the feedstock [54,67,70]. Filament-based systems require adequate filament dimensions, mechanical strength and feeding behaviour, whereas semi-solid extrusion depends on formulation rheology, shape retention and any subsequent drying or curing stage [54,67]. Claims concerning the advantages or limitations of additive manufacture should consequently remain platform-specific, as thermal exposure, solvent requirements, material compatibility, dimensional accuracy, layer adhesion and post-processing cannot be generalised across printing routes [54,67,70].

No manufacturing strategy can, therefore, be considered intrinsically preferable independently of formulation and product requirements, as each route replaces one group of processing constraints with another [47,52,55]. Solvent casting places particular emphasis on liquid-formulation and drying control, hot-melt extrusion on thermomechanical processability, electrospinning on fibre formation and environmental conditions, and additive manufacturing on platform-specific feedstock and deposition behaviour [53,54,62,63].

### 2.2. Film Architecture and Antimicrobial Drug-Loading Strategies

**Figure 1:**
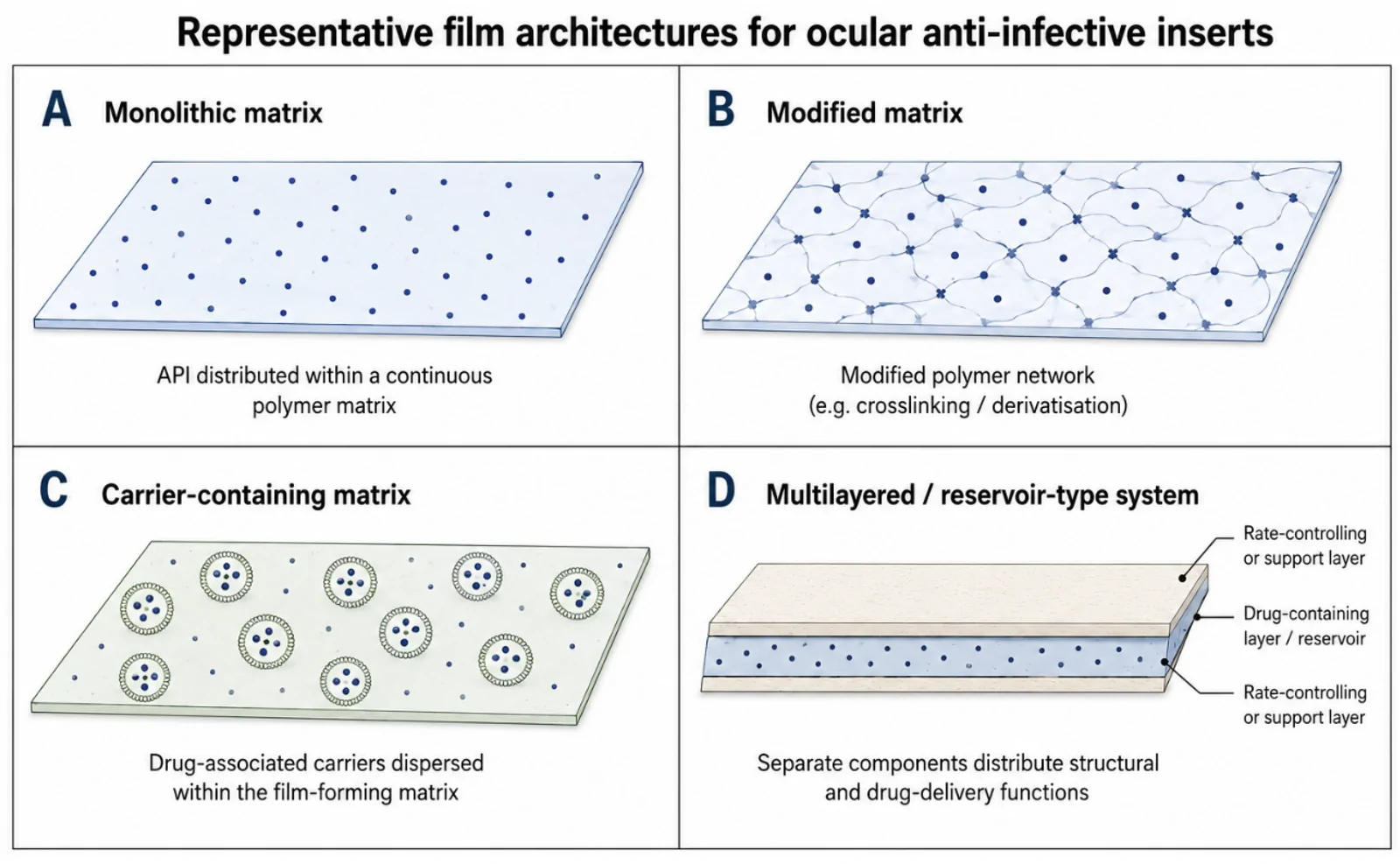
Representative film architectures investigated for ocular anti-infective inserts. Schematic overview of the principal structural configurations used to incorporate and deliver an active pharmaceutical ingredient (API) from polymeric ocular thin film inserts. (**A**) Monolithic matrix: API dissolved, dispersed or otherwise incorporated throughout a continuous polymer matrix. (**B**) Modified matrix: API distributed within a polymer network whose structure or chemical properties have been altered. (**C**) Carrier-containing matrix: API associated with discrete drug carriers dispersed within the surrounding film-forming matrix. (**D**) Multilayered or reservoir-type system: insert comprises spatially separated layers with distinct structural and drug-delivery functions; drug-containing layer or reservoir positioned between, or adjacent to, rate-controlling or support layers. [Illustrated categories are not mutually exclusive and not to scale].

The location of the API relative to the polymeric components provides a functional basis for classifying ocular inserts, as it defines which part of the dosage form contributes to drug loading, structural integrity and control of release [1,46,61]. Reported systems can be grouped broadly as monolithic matrices, modified matrices, carrier-containing matrices and multilayered or reservoir-type systems, while recognising that individual formulations may combine features of more than one category [1,46,61]. This classification is illustrated in Figure 1.

Monolithic matrices contain the API within a continuous polymeric phase following molecular dissolution, dispersion or separate solubilisation before incorporation [48,57,71]. Their structural simplicity avoids preparation and assembly of separate components, although the absence of distinct layers does not imply simple release behaviour, which remains dependent on the physical state of the API, polymer–solvent compatibility and matrix hydration, swelling, erosion or dissolution [47,48,57]. Formulation changes intended to retard drug transport, including increased polymer concentration or incorporation of less permeable constituents, may also alter precursor viscosity, film thickness, flexibility and the proportion of drug recovered during release testing [48,49,57]. The relative simplicity of the architecture should therefore be distinguished from the number of formulation variables capable of influencing its performance [47,48].

Modified matrices retain a continuous polymeric phase but incorporate physical or chemical alteration of the polymer network, including ionic crosslinking, covalent crosslinking or derivatisation, to modify hydration, mucoadhesion, structural integrity or drug release [1,59,72]. Calcium-treated or thiolated alginate and crosslinked gelatin provide examples in which the characteristics of the resulting film depend on both the original polymer and the conditions used to modify it [59,73,74,75]. The effects observed for these systems cannot therefore be attributed to polymer identity alone, as reagent concentration, treatment duration, temperature, washing and the achieved extent of modification may influence matrix behaviour and drug accessibility [1,59,72]. Interpretation consequently requires explicit reporting of the modification procedure and evidence of the resulting change in polymer or film characteristics [1,59,72].

Carrier-containing matrices incorporate drug-associated nanoparticles, liposomes or molecular complexes within a surrounding film-forming phase [61,67,76]. This configuration separates, to some extent, the immediate environment of the API from the polymeric matrix, potentially allowing drug association and carrier-mediated release to operate alongside hydration and erosion of the film [61,76]. The additional structural component also introduces requirements for carrier loading, stability and distribution within the precursor formulation and finished insert, which should be considered together with film thickness, mechanical characteristics and overall drug-content uniformity [61,76]. Where an otherwise equivalent film containing a non-associated drug is not evaluated, the respective contributions of the carrier and surrounding matrix cannot readily be separated, limiting conclusions concerning the functional advantage of the combined system [61].

Multilayered and reservoir-type systems distribute drug-delivery and structural functions among separate components, commonly through a drug-containing layer or compartment combined with supporting, less permeable or rate-controlling layers [56,58,60]. This arrangement may support directional, sequential or biphasic release, although the intended contribution of each layer remains dependent on reproducible control of composition, individual and total thickness, interlayer adhesion and structural integrity during hydration [58,59,60]. Sequential casting, coating or lamination may also expose an earlier layer to additional solvent or processing conditions, creating the possibility of intermixing or alteration of the previously formed component [56,58,60]. The added architectural complexity should, therefore, be linked to a demonstrable contribution of the separate layers rather than inferred from prolongation of release alone [49,56,58].

Architecture selection cannot be separated from the physicochemical characteristics of the API, as aqueous solubility, ionisation, solid-state form, thermal stability and interactions with polymers or carriers can affect incorporation, distribution, stability and release [47,52,57]. An API that remains molecularly dissolved within a monolithic matrix introduces different risks from a dispersed drug or carrier-associated system, particularly as solvent removal, thermal exposure or hydration may alter drug distribution or physical state [57,61,77]. The architecture should consequently be selected in relation to the state in which the API can be incorporated and retained, rather than according to release duration alone [47,57,61].

### 2.3. Polymer Selection, Blending and Plasticisation

The essential formulation components of an ocular anti-infective thin film insert are the API and at least one polymer capable of forming a discrete matrix [46,47]. Secondary polymers, plasticisers and other excipients may be incorporated to adjust processability, mechanical behaviour, API incorporation, hydration, surface interaction or drug transport according to the requirements of the formulation [49,78,79]. These attributes are interdependent because molecular interactions that strengthen the dried matrix may also increase precursor viscosity or restrict API movement, whereas greater chain mobility or water affinity may improve flexibility and drug liberation while reducing dimensional stability or matrix-mediated control [50,78,80,81]. Selection should therefore be based on the connected behaviour of the finished insert during manufacture and use, rather than on the generic identity of a polymer or a favourable mechanical, swelling, adhesion or release result considered independently [46,48,49]. Table 2 summarises the material-specific evidence supporting this analysis.

Polymer identity provides only a broad indication of formulation behaviour because the relevant molecular characteristics vary with material grade. Molecular weight and chain architecture influence entanglement and precursor viscosity, while substitution, hydrolysis or deacetylation determine the density and accessibility of groups involved in water association, hydrogen bonding, protonation or ionic coordination [82,90,92,106]. Ionic form, molecular-weight distribution and biological source may introduce additional variability in the organisation and aqueous response of polysaccharide- or protein-based materials [92,102]. These characteristics affect how readily chains associate during film formation and separate after water uptake, with consequences for rheology, matrix cohesion, swelling, erosion and resistance to drug movement [80,81,82,106]. Grades reported under the same generic polymer name should not therefore be treated as interchangeable, particularly where viscosity grade, substitution, hydrolysis, deacetylation or source has not been specified adequately [82,92,102,106]. Incomplete material specification limits causal interpretation of the reported formulation behaviour and reduces the likelihood that dosage-unit uniformity, hydrated performance and drug liberation can be reproduced across batches, laboratories or APIs [47,48,49].

Polymer concentration, blend ratio and plasticisation determine how these grade-specific characteristics are expressed within the formulation. Increasing polymer content raises the frequency of chain contact and may improve cohesion after drying, although the accompanying increase in precursor viscosity can hinder mixing, deaeration, levelling, mould filling or casting [47,57,117]. PVA/Soluplus^®^ systems illustrated this processing boundary, as increasing polymer content eventually produced precursor formulations that were unsuitable for satisfactory casting [57]. Increasing guar-gum concentration produced a different limitation, with greater swelling and tensile performance accompanied by lower drug permeation across excised cornea under the applied conditions [105]. Although the studies were not directly comparable, both indicated that reinforcement of the polymer network may narrow the processable range or increase resistance to drug movement [57,105]. The useful concentration range should consequently support homogeneous API distribution and reproducible unit formation without allowing an improvement in dry-film strength to obscure a loss of processability or drug transport [47,49].

Blending may extend this range where the principal film-forming polymer does not provide an adequate balance of cohesion, hydration, adhesion or drug transport. A second polymer may be incorporated to increase water association, introduce ionisable or mucoadhesive groups, modify chain packing or regulate diffusion, while the principal polymer preserves continuity of the dosage unit [79,81]. The anticipated benefit depends on compatible interactions between the selected grades and on a blend ratio that supports the intended function without producing excessive viscosity, phase heterogeneity or resistance to API movement [49,79,81]. Incorporation of CMC or gelatin into PVA-containing matrices modified release and adhesive or mechanical behaviour, which supported a blend-level effect but did not isolate a universally favourable contribution from either secondary polymer [74,87]. Attribution was also restricted in the reported PVA–PVP series because polymer proportions, plasticiser content and the accompanying Eudragit^®^ grade varied concurrently [108]. A defensible rationale for blending should therefore identify the deficit in the initial matrix, the interaction expected to address it and the compositional range over which the intended improvement remains reproducible [79,81]. Evidence of improvement in one endpoint does not demonstrate complementarity where the same blend impairs formulation rheology, dosage-unit uniformity, hydrated integrity or total drug recovery [47,49,50].

Plasticisers modify the same network by increasing segmental mobility and reducing or redistributing association between polymer chains [78,113]. This may reduce brittleness and facilitate film removal, subdivision, packaging and manipulation, while the accompanying increase in free volume can also promote water penetration, matrix permeability and API diffusion [78,113]. Faster antibiotic release at higher PEG 400-to-polymer ratios across several polymer matrices indicated that changes attributed to polymer concentration may partly arise from simultaneous variation in relative plasticisation [94,99]. Plasticiser identity, molecular grade, concentration, compatibility and distribution should consequently be related to both dry-state mechanics and performance following hydration [78,114,115]. Greater flexibility may not provide a formulation advantage where it is accompanied by tack, migration, uncontrolled dimensional change or loss of drug-transport control during storage or use [78,114,115]. The selected level of plasticisation should support reproducible manufacture and manipulation of the insert while preserving its hydrated integrity and intended release behaviour [25,46,49]. Historical use of dibutyl phthalate demonstrated technical functionality in ocular inserts but provides limited precedent for new formulations in view of wider phthalate-related toxicological concerns [75,84,116].

Upon administration, water penetrates the film and redistributes the polymer–polymer and polymer–API associations established during drying [80,82,106]. Water uptake is promoted by accessible polar or ionisable groups, whereas chain entanglement, ordered association and ionic or covalent junctions resist separation of the hydrated chains [80,92,106]. The balance between these effects determines whether the insert swells while remaining cohesive, develops a gel layer, progressively erodes, dissolves or persists as an extensively hydrated structure [46,80]. Rapid hydration may facilitate early API liberation but can also produce deformation or premature loss of integrity, whereas a more resistant network may preserve the dosage unit while increasing diffusional resistance or retaining part of the incorporated dose [46,50,80]. Hydrophilicity, swelling and nominal aqueous solubility should therefore be interpreted through their combined effects on hydrated dimensions, mechanical behaviour, drug recovery and the amount and form of material remaining during administration [25,46,48,49]. Mechanical suitability in the dry state does not establish that the insert will retain appropriate geometry or integrity after tear uptake, supporting assessment of the administered dosage form under hydrated conditions [25,46,49].

The aqueous conditions used during formulation testing influence how these molecular relationships are expressed. Bulk immersion may expose the insert to a substantially larger and less dynamic fluid environment than the restricted and continuously renewed tear film [46,48,49]. Medium composition, pH, ion availability, volume, renewal and hydrodynamic conditions may alter polymer ionisation, ionic association, swelling, erosion and API transport [89,90,91,111]. These variables are particularly relevant to matrices relying on calcium-mediated junctions or pH-responsive components, whose behaviour may change with the chemical environment surrounding the insert [89,90,91,111]. A slowly transforming matrix under bulk-immersion conditions may not preserve the same balance between hydration, erosion and release following conjunctival administration [46,49]. Interpretation of aqueous performance should therefore account for both the test conditions and the molecular mechanism used to regulate matrix transformation [48,49].

Hydration also changes the mobility and accessibility of groups capable of interacting with ocular-surface mucin. Accessible hydroxyl and carboxyl groups may support hydrogen bonding, while protonated amino groups may provide electrostatic association under suitable pH and ionic conditions [92,95,97]. The resulting interaction depends on molecular weight, charge density and hydrated-chain mobility rather than on the presence of the relevant functional group alone [92,95]. Blending, crosslinking or coating may restrict chain movement or cover the film surface, reducing access to groups expected to support adhesion [75,81]. Calcium treatment and subsequent Eudragit^®^ coating of PVA–alginate inserts extended release while progressively reducing measured ex vivo mucoadhesion, indicating that the same interventions that restricted transport also altered the surface available for mucosal interaction [75]. The goat-eyelid method did not establish in vivo positioning, residence or comfort, but the comparison identified a release–surface-interaction trade-off that could not have been predicted from the nominally mucoadhesive constituents alone [25,46,75]. Mucoadhesion should accordingly be considered with hydrated dimensions and mechanics, as the highest measured adhesion does not independently establish reliable or acceptable ocular residence [25,46,49].

API liberation reflects both the physicochemical state of the drug and the changing organisation of the hydrated polymer network. Drug dissolution, diffusion through water-filled regions, polymer relaxation, matrix erosion and drug–polymer association may operate concurrently, with their relative contribution changing throughout hydration [50,80]. In calcium-treated alginate inserts, crystalline ciprofloxacin was released more slowly than its hydrochloride form, while HPMC incorporation produced additional prolongation for both API forms [73]. Because the same selected calcium treatment was applied, the observed differences could not be attributed to the alginate network alone and instead reflected the combined influence of API dissolution and transport through the HPMC-containing phase [73]. Tear-concentration measurements in rabbits extended interpretation beyond the release profile by relating local exposure from the crystalline formulations to the stated antimicrobial susceptibility benchmark [73]. This evidence was more informative for anti-infective delivery than prolonged release alone because it addressed whether the amount liberated remained microbiologically relevant during the evaluated period [1,73]. Where comparable exposure–activity evidence is unavailable, release duration should be interpreted with total API recovery and preservation of antimicrobial activity, as continued retention within the matrix does not establish prolonged therapeutic availability [1,48,50].

Additional resistance to hydration or drug transport can be introduced through ionic coordination, covalent hardening or a separate rate-controlling layer. These approaches share the objective of modifying network permeability, but differ in reversibility, manufacturing control and the amount or type of material that may remain after release [90,102,111,112]. Calcium coordination produces reversible junctions between alginate carboxylate-containing regions and remains dependent on alginate composition, ion availability and treatment conditions [90,91]. Glutaraldehyde hardening introduces covalent associations within gelatin-containing matrices and requires control of reagent exposure, neutralisation, washing and residual crosslinker [71,102]. Higher gelatin concentration following equivalent hardening was associated with longer release, but the absence of an uncrosslinked comparator prevented the effects of polymer content and covalent modification from being separated [71]. Residual glutaraldehyde was not quantified despite the reported neutralisation and washing procedures, leaving a safety-relevant characteristic of the processed material unresolved [71,102]. Chemically modified matrices should therefore be compared with an otherwise equivalent unmodified formulation and characterised for residual components relevant to ocular exposure as well as their intended mechanical or release effect [49,59,102].

Rate-controlling membranes alter transport through the chemistry and structure of a separate external layer. Poorly water-soluble barriers regulate transport principally through limited aqueous affinity and permeability, whereas ionisable coatings undergo pH-dependent changes in solubility or permeability [89,111,112]. Comparisons among membranes applied to similar drug-containing matrices supported an effect of barrier chemistry but did not establish the least permeable or slowest-releasing system as the most appropriate architecture [56,75]. Thickness, continuity, plasticisation and interlayer adhesion may modify membrane behaviour independently of nominal polymer identity and should be specified together with the properties of the underlying reservoir [49,58,60]. Prolonged transport should also be interpreted with total drug recovery, hydrated flexibility and the subsequent behaviour of any poorly soluble layer [25,46,49]. The added complexity of a multilayered system may be justified only where it provides reproducible active-drug exposure without producing a poorly integrated, inflexible or unnecessarily persistent dosage form [25,46,49].

The same critical principle applies where the polymer contributes biological activity or modifies epithelial transport. Intrinsic antimicrobial activity, as reported for chitosan, may contribute to microbial inhibition independently of the incorporated API and should be controlled using an otherwise equivalent drug-free matrix [93,94]. Any increase in epithelial drug passage should be assessed with barrier integrity and cytotoxicity, as enhanced permeation may not represent an advantage where it reflects ocular-surface disruption [19,20]. Chitosan was not represented in a recent FDA-IID-based compilation of excipients used in approved human ophthalmic products, which does not establish ocular unsuitability but limits the extent to which experimental use can be regarded as established product precedent [100]. Comparable scrutiny is required for biologically sourced or derivatised polymers, plasticisers, residual crosslinkers and persistent membrane components because favourable film formation, adhesion or release does not independently establish acceptability of the processed material for ocular exposure [49,102,116].

Selection of the polymeric system should progress from a defined material specification to evaluation of the interactions introduced by concentration, blending, plasticisation and processing [47,49,81]. Those interactions should then be assessed within the hydrated and drug-releasing insert, where aqueous transformation, surface behaviour and API availability become directly relevant to use [1,46,49]. A suitable composition should remain processable and reproducible and provide sufficient integrity for manipulation and placement [25,47,49]. It should also transform predictably following tear exposure, release microbiologically active API and leave an acceptable amount and form of material at the ocular surface [1,46,48]. Evaluation of these connected outcomes provides a more rigorous basis for material selection than the generic polymer name, an isolated physicochemical measurement or the duration of an in vitro release profile [46,48,49].

### 2.4. Solvent-Casting Process Variables

**Figure 2:**
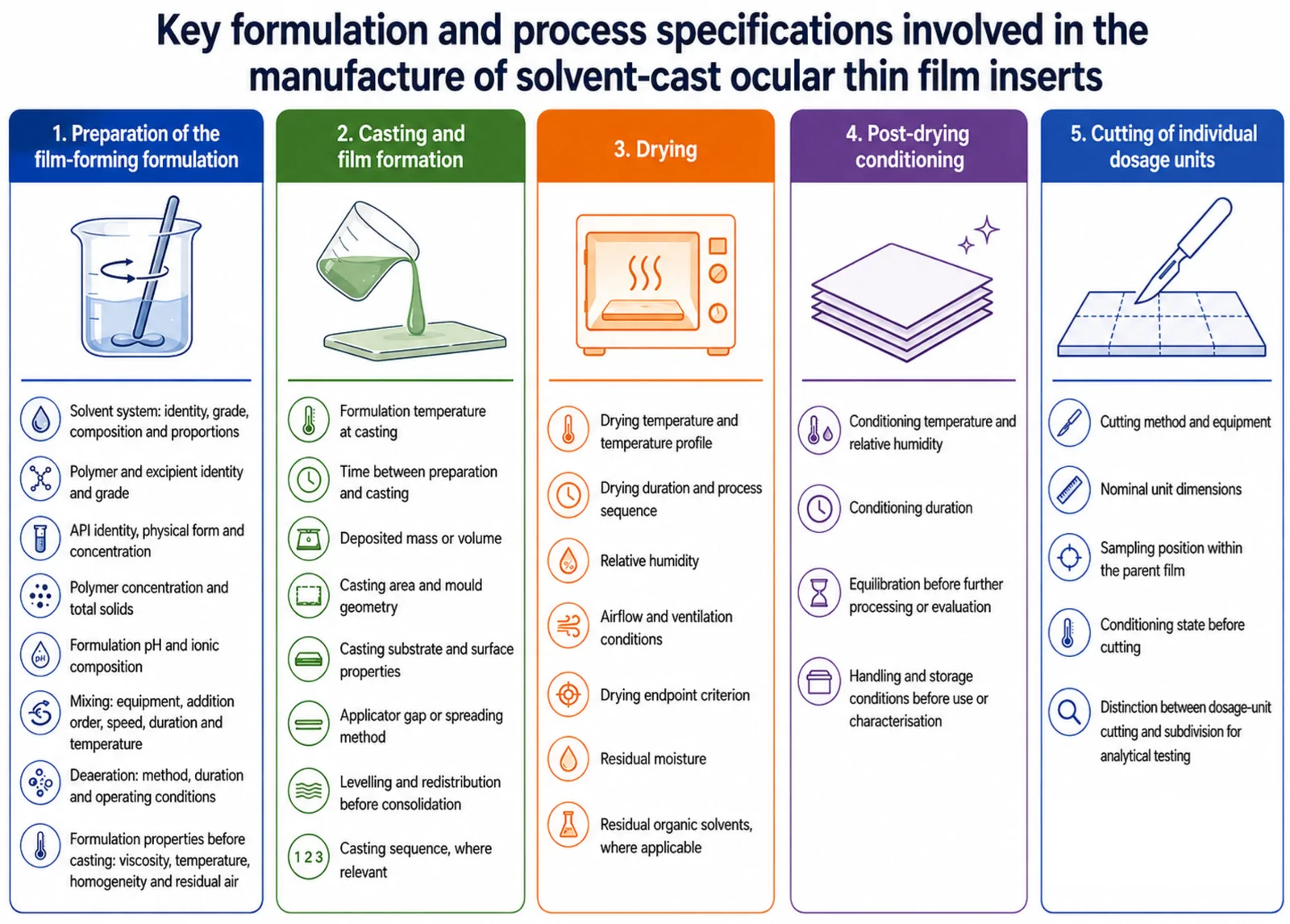
Key formulation and process specifications involved in the manufacture of solvent-cast ocular thin film inserts. Schematic overview of the principal formulation attributes and processing variables that should be defined, controlled and reported during solvent-casting manufacture. 1. Preparation of the film-forming formulation: solvent, polymer, excipient and API characteristics, formulation composition, mixing, deaeration, and properties before casting. 2. Casting variables: formulation temperature, time before casting, deposited quantity, casting geometry, substrate, spreading method, and film levelling. 3. Drying conditions and endpoints: temperature, duration, humidity, airflow, residual moisture, and residual organic solvents, where applicable. 4. Post-drying conditioning: equilibration, handling, and storage conditions before further processing or characterisation. 5. Individual dosage units: cutting method, unit dimensions, sampling position, and conditioning state before cutting. Applies only to manufacturing approaches in which a parent film is cut or subdivided.

The characteristics of the polymeric system shape the behaviour of the finished insert and the conditions under which the formulation can be prepared, deposited and converted into a coherent film, as polymer identity, grade, concentration and blending influence solvent compatibility, viscosity, thermal behaviour and matrix formation [47,81,118]. These formulation–process relationships are particularly relevant to ocular anti-infective inserts, for which solvent casting remains the most frequently reported manufacturing route [48,51,57,61]. Solvent composition and total solids influence polymer hydration and formulation rheology, with subsequent effects on mixing, deaeration and layer formation, while deposition and drying determine removal of the processing medium and consolidation of the polymeric matrix [77,118,119]. Preparation, casting and drying therefore form a connected manufacturing sequence, as changes introduced during formulation preparation may remain evident in film dimensions, drug distribution, mechanical behaviour, residual moisture or solvent and release characteristics [118,120,121]. The principal formulation and process specifications requiring definition across solvent-cast manufacture are summarised in Figure 2.

#### 2.4.1. Preparation of the Film-Forming Formulation

Selection of the processing medium should support incorporation of the API, polymers and accompanying excipients as a sufficiently uniform solution, suspension or carrier-containing dispersion, while providing rheological characteristics compatible with transfer and casting and permitting adequate removal during drying [77,118,119]. Aqueous formulations predominated among monolithic ocular anti-infective films, consistent with the frequent use of hydrophilic polymeric systems, whereas mixed aqueous-organic media were employed in some reservoir and multilayered formulations containing components with differing solubility characteristics [58,60,83]. Initial solubility provides only one aspect of solvent-system suitability, as solvent composition can also influence polymer association and the physical state of the API during the progressive increase in drug and polymer concentration associated with drying [77,118,122]. The pH and ionic composition of the medium introduce additional considerations where polymer hydration, association or gelation is sensitive to the surrounding environment [75,105,123]. Buffered media were used in some ocular-film formulations, although their influence on formulation state and processability was not always characterised independently [75]. Where ionic or pH-responsive behaviour forms part of the intended post-administration performance, its occurrence during preparation could alter viscosity or matrix organisation before casting, indicating that solvent composition, pH and ionic strength should be considered in relation to both component incorporation and maintenance of a processable formulation state [75,105,118,123].

The requirements imposed on the processing medium depend partly on whether the API is molecularly dissolved, dispersed as a solid phase or associated with a carrier [57,61,77]. Dissolved APIs may undergo changes in physical state as solvent removal increases drug and polymer concentrations, including phase separation or recrystallisation where the evolving matrix no longer maintains the original state [77]. Dispersed APIs and drug-containing carriers require sufficient physical stability to limit sedimentation, aggregation or redistribution before the polymeric matrix has consolidated [57,61]. Solvent-system suitability should consequently be considered according to whether the intended API state and distribution can be maintained throughout preparation, casting and drying, rather than according to initial incorporation alone [57,61,77].

Where organic or mixed aqueous-organic media are required, solvent selection should account for API and polymer solubility, compatibility among the formulation components, solvent volatility and the feasibility of removing the processing medium under conditions compatible with API and matrix stability [77,118]. The composition of a mixed-solvent system may evolve during drying as its constituents evaporate at different rates, so the solvent environment governing polymer association and API state may differ progressively from that present during formulation preparation or casting [77,121]. Greater volatility may shorten the drying period, although evaporation rate remains linked to deposited-layer thickness, matrix consolidation and the extent of volatile material retained within the resulting film [77,121]. Solvent identity, grade, composition and relative proportions should, however, be reported together with the drying conditions and any solvent-specific assessment applied to the finished film [118].

Formulation viscosity provides a direct connection between polymer-system selection and processability, as it is influenced by polymer identity, molecular grade, concentration, total solids, temperature and interactions among the constituent materials [47,48,118]. Low viscosity of the film-forming solution may permit sedimentation, phase separation or excessive spreading, particularly where the API or a carrier remains dispersed, whereas high viscosity may impede polymer hydration, component incorporation, formulation transfer, removal of entrained air and levelling after deposition [48,105,118]. Polymeric systems selected for viscosity enhancement, gel formation or adhesion may consequently provide relevant post-administration characteristics while imposing more demanding preparation and casting conditions [13,48,105]. A formulation sufficiently viscous to limit movement of suspended material may be more difficult to deaerate or level after deposition, while one that spreads readily may provide less resistance to redistribution before drying [105,124]. Interpretation also depends on the measurement conditions, since polymeric formulations may exhibit shear- and temperature-dependent behaviour that cannot be inferred from polymer concentration alone [117,118]. Viscosity should therefore be evaluated within a formulation-specific processing range and reported together with the temperature and applied measurement conditions needed to relate the value to the preparation and casting operations used [48,117,118].

Mixing influences polymer hydration or dissolution, incorporation and distribution of the API and excipients, and the amount of air introduced into the formulation [118,119,120]. Mixer configuration, vessel geometry, component-addition sequence, operating speed, duration and temperature can each influence the state of the formulation supplied for casting [118,119,120]. In pharmaceutical polymer films containing low drug quantities, variation in mixing conditions affected drug distribution and content uniformity, indicating that apparent homogeneity of the liquid formulation does not necessarily establish uniformity in the dried film [120]. Mixing and homogenisation conditions have also been associated with changes in physicochemical, mechanical and barrier characteristics in solvent-cast systems, although the direction and magnitude of these effects remained formulation-specific [125,126]. Heating may facilitate polymer hydration or dissolution and alter viscosity through changes in polymer–polymer and polymer–solvent interactions, with temperature-dependent hydrogen bonding and molecular mobility being particularly relevant to PVA-containing aqueous systems [81,117]. Since blending can modify the hydration and thermal behaviour of the constituent polymers, preparation conditions should be selected for the complete formulation while accounting for the stability of heat-sensitive APIs and excipients [81,127].

Considerable variation was evident in the procedures reported for ocular anti-infective films, with mixing durations extending from approximately 30 min to 12 h and stated speeds ranging from 100 to 700 revolutions per minute, while other formulations relied on qualitative endpoints such as complete dissolution or apparent homogeneity [57,73,74,87,105,108,128]. Mixing temperatures were described principally for PVA-based ocular films and selected hydrogel-forming systems and were less consistently reported for other polymer combinations [74,87,109,129]. The reported ranges do not support identification of a universally preferable procedure, since the required conditions remain dependent on polymer composition, formulation volume, equipment and temperature [118,120]. Incomplete specification nevertheless limits assessment of whether differences attributed to composition or drug loading may also have reflected variation in polymer hydration, component distribution or air incorporation [118,120].

Air introduced during polymer hydration and mixing may persist within viscous formulations and contribute to pores, discontinuities or surface defects, while prolonged agitation cannot be assumed to provide effective deaeration and may introduce further air [118,119]. Reported approaches included standing the formulation for periods ranging from several hours to overnight, bath sonication and centrifugation followed by removal of visible bubbles [48,98,105,123]. Their suitability remains dependent on formulation viscosity and physical stability, since prolonged standing may permit sedimentation or phase separation, while high viscosity can restrict bubble movement and removal [105,118]. The formulation supplied for casting should consequently be defined by its composition, temperature, viscosity, homogeneity and residual air content [118,120].

#### 2.4.2. Casting and Film Formation

Casting converts the prepared formulation into a layer of defined area and nominal composition before removal of the processing medium [47,118]. The dimensional and compositional characteristics established during this stage remain dependent on the deposited quantity, casting area, formulation rheology and redistribution occurring before consolidation [118,130]. Ocular anti-infective films have been prepared either by dispensing the formulation into individual moulds or by casting a larger sheet subsequently divided into dosage units [47,48]. Individual moulds define the nominal area of each unit before drying, whereas larger sheets require adequate spatial uniformity across the parent film and appropriate selection of the regions used for subdivision [118,130]. In a non-ocular polymeric-film system, use of a silicone tray containing individual moulds reduced variability in thickness, drug content, release and mucoadhesion relative to continuous-tray casting [130]. Although differences in formulation and intended use prevent generalisation to ocular anti-infective inserts, the findings illustrate that casting geometry can influence unit uniformity independently of nominal composition and should therefore be reported as part of the manufacturing procedure [118,130].

The casting substrate may affect formulation spreading, adhesion, heat transfer and detachment of the dried film, with its suitability dependent on surface smoothness, level, dimensional stability and compatibility with the formulation and solvent system [118,131]. Glass Petri dishes were frequently used as moulds, while glass, plastic and metal surfaces were also employed for preparation of larger films or subsequent heated drying [48,58,59,83]. No consistent relationship was evident between substrate material and drying procedure, indicating that material type alone provides limited information regarding its influence on film formation [48,118]. Mercury was used in some formulations to provide a level casting interface, occasionally with rings positioned on the liquid metal to define individual units [56,99,105]. Although this configuration provided a fluid and level surface, the toxicity and handling and disposal requirements associated with mercury substantially limit its relevance to contemporary pharmaceutical-film manufacture [131]. Reporting should therefore identify the actual substrate and casting configuration rather than referring only to a dish, tray or mould [118,131].

Formation of a uniform layer depends on the combined effects of deposited mass or volume, casting area, formulation solids, substrate level and rheological behaviour [118,119]. High-viscosity formulations may resist spreading and retain local thickness differences, whereas lower-viscosity systems may spread beyond the intended area or permit movement of dispersed APIs or carriers before consolidation [57,118]. Incomplete transfer and non-uniform levelling may consequently produce local differences in thickness, composition or surface appearance despite apparent homogeneity of the precursor formulation [48,118,124]. Adjustable applicators can regulate the distance between the spreading blade and substrate where unrestricted spreading provides insufficient dimensional control [118,131]. The selected applicator gap should not be equated directly with final film thickness, since formulation solids, completeness of transfer, levelling and contraction during drying influence the resulting dimensions [77,118]. Deposited quantity, casting area, formulation solids, applicator gap and measured film thickness should therefore be reported as related but non-equivalent manufacturing and product attributes [118,119,130].

The interval between formulation preparation and deposition may alter the material delivered to successive moulds or different regions of a larger sheet, particularly where dispersed components can sediment or aggregate, volatile constituents can evaporate, or polymer behaviour changes with temperature [57,118]. Formulation temperature, elapsed time before deposition and casting sequence should therefore be reported where these variables may influence film composition, dimensions or surface characteristics [77,118].

#### 2.4.3. Drying, Post-Drying Conditioning and Cutting

Drying should be considered an integral step in the matrix-forming process, rather than a simple solvent-removal step, as progressive concentration of the formulation accompanies polymer consolidation, redistribution of components, and potential changes in API and excipient organisation [77,121]. Interpretation of the process requires, therefore, at least reported temperature and duration, since evaporation and matrix formation are also influenced by solvent composition, deposited-layer thickness, airflow, relative humidity and the mobility of the processing medium within the increasingly concentrated polymeric phase [47,77,121]. These variables have not been consistently described for ocular anti-infective films, limiting comparison among procedures that appeared similar on the basis of nominal temperature and drying time [47,121].

Drying temperature cannot be interpreted independently of formulation composition and environmental conditions, as accelerated evaporation may alter matrix contraction, plasticiser retention, surface formation, and mechanical behaviour, as well as shortening the drying period [121,132,133]. This dependence was illustrated by alginate-containing films in which increasing temperature was associated with changes in thickness, elongation, retained glycerol, tensile strength and surface appearance, although the specific outcomes remain confined to the formulations and drying environments examined [132,133]. Such findings, however, support the need to evaluate the resulting film characteristics alongside drying efficiency, rather than treating a shorter process as inherently preferable [121,132,133].

The reported use of ambient, heated and staged drying procedures appears to reflect adaptation to different polymer and solvent systems, but the absence of consistently defined drying endpoints restricts interpretation of their relative suitability [58,59,83,84,128]. Elevated temperatures were frequently used with organic or mixed-solvent formulations, while some systems underwent additional room-temperature drying after heated treatment and others containing predominantly aqueous media were also dried under elevated temperature [58,59,83,84,109]. These procedures cannot be compared meaningfully without appraising the interplay with solvent composition, layer thickness, airflow, relative humidity, and the criterion used to establish completion of drying [47,48,121]. In addition, the completion of film formation should be distinguished from completion of solvent or water removal, as visual coherence does not establish the amount or state of volatile material remaining within the matrix [118,121]. Changes among free, bound and clustered water during drying of sodium alginate formulations provide an example of how internal moisture can persist after the surface has formed and the matrix appears dry [134]. Predetermined drying periods and mass stabilisation may indicate that further overall volatile loss has become limited under the applied conditions, but neither approach identifies the retained components or establishes their suitability for the intended dosage form [121].

Residual water and residual organic solvents require separate analytical consideration, as their functional significance and acceptable levels are not equivalent [118]. Water retained within hydrophilic matrices may influence plasticisation, flexibility, molecular mobility, and subsequent sensitivity to environmental humidity, while extensive removal may favour increased rigidity or brittleness [132,134]. Residual organic solvents, instead, require solvent-specific assessment according to their identity, toxicological classification and permitted exposure, and this distinction is also particularly important for mixed-solvent systems, in which individual components may evaporate and be retained to different extents, so gravimetric loss or mass stability cannot demonstrate the residual concentration of each solvent [118,121]. Analytical procedures should consequently be selected according to the solvent system and reported together with the drying conditions and endpoint applied [118].

The physical state of the film may continue to evolve after removal from the drying environment, particularly where hygroscopic polymers or hydrophilic plasticisers exchange moisture with the surrounding atmosphere [121,132]. Conditioning under defined temperature and relative-humidity conditions can therefore provide a more consistent basis for detachment, subdivision and characterisation, whereas uncontrolled exposure may alter thickness, flexibility, mechanical behaviour and measured moisture content before testing [121,132]. The duration and sequence of drying, conditioning and subsequent handling should, consequently, be specified where these operations may affect comparison among specimens or formulations [121].

Cutting represents the final conversion of a larger cast film into individual dosage units and should be distinguished from subdivision performed solely to obtain analytical specimens [118,130]. Assignment of mass and nominal drug content according to unit area assumes adequate spatial uniformity of thickness and composition across the parent film, so identical dimensions do not necessarily establish equivalent dosage units [118,130]. The cutting method, nominal dimensions, sampling position and conditioning state before subdivision should therefore be reported where these factors may influence subsequent physicochemical, mechanical or release measurements [118,130].

## 3. Testing Methodologies

Methods used to characterise ocular films remain heterogeneous, with substantial differences in the attributes examined, experimental conditions applied and criteria used to interpret performance [135,136]. This variability restricts comparison among formulations and complicates identification of the evidence required to support progression from pharmaceutical development towards clinical evaluation. Although regulatory approval has been achieved for other ophthalmic drug-delivery devices, including a drug-eluting contact lens, an equivalent, comprehensive evaluation framework has not been established for ocular inserts, whether film-based or otherwise [29,137].

General pharmacopoeial requirements for ophthalmic preparations and uniformity of dosage units provide relevant reference points, although no complete insert-specific framework—whether for film-based or other ocular inserts—defines the full set of methods and acceptance criteria required for medicated ocular thin films [135,138,139]. Methods have consequently been adapted from conventional solid dosage forms, polymeric films, buccal products, and other ophthalmic preparations, with considerable variation in specimen preparation, sample number, apparatus, exposure conditions and reporting [47,49,136]. Rather than imposing a single universal procedure across all formulations, standardisation could combine a consistent core assessment of dosage-unit quality, physical integrity, drug content, sterility and ocular compatibility with formulation-specific evaluation of hydration, matrix transformation, drug release and antimicrobial performance [46,136].

**Figure 3:**
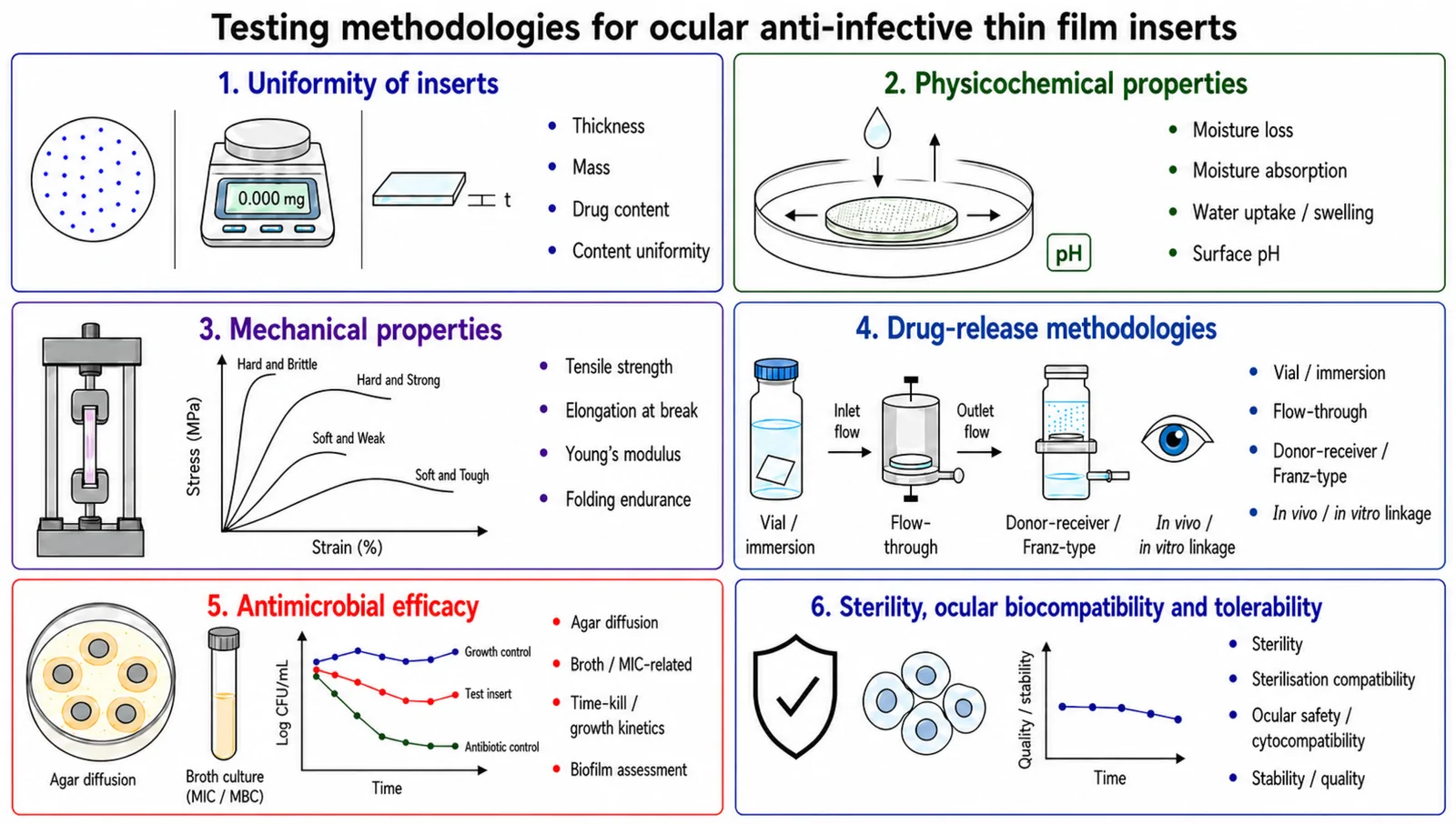
Testing methodologies for anti-infective thin film inserts. Schematic overview of the principal experimental approaches used to characterise ocular anti-infective thin film inserts. 1. Uniformity of inserts: physical and pharmaceutical consistency across individual inserts, including thickness, mass, drug content and content uniformity. 2. Physicochemical properties: interaction with moisture and aqueous environments through measurements of moisture loss, moisture absorption, water uptake or swelling, and surface pH. 3. Mechanical properties: structural and handling-mimicking behaviour, using tensile testing and complementary measures of mechanical durability. 4. Drug-release methodologies: drug liberation and release, including vial or immersion testing, flow-through systems, and donor–receiver or Franz-type diffusion arrangements, and the need to relate findings obtained under in vitro conditions to in vivo performance. 5. Antimicrobial efficacy: antimicrobial activity against relevant microorganisms, including agar-diffusion assays, broth-based minimum inhibitory concentration or minimum bactericidal concentration testing, time-kill or growth-kinetic analysis. 6. Sterility, ocular biocompatibility and tolerability: sterility assurance and compatibility, ocular safety or cytocompatibility, and stability-related quality attributes over time.

The following section evaluates the procedures reported for anti-infective ocular films across dosage-unit uniformity, physicochemical and mechanical characterisation, drug release, antimicrobial activity, sterility assurance, and ocular biocompatibility and tolerability, as summarised in Figure 3. The discussion aims to define current practices, identify methodological limitations and support more reproducible and clinically informative evaluation of ocular thin film inserts. Methodologies used for pharmaceutical films administered by other routes provide useful points of reference, although their direct transfer to ocular inserts requires consideration of the administration site, limited fluid availability, matrix behaviour and intended duration of exposure [135,136].

### 3.1. Uniformity of Inserts

Ocular film inserts constitute single-dose preparations, irrespective of whether the units are cast individually or cut from a larger parent sheet, and formulations should, therefore, satisfy appropriate drug-content uniformity criteria, which may be evaluated using different analytical approaches [140,141]. Although the amount of drug incorporated into each insert represents a fundamental formulation attribute [50], the procedures applied to assess uniformity in polymeric films have not always provided adequate characterisation [48,130].

Thickness and mass are routinely measured during film characterisation [142]. In film formulations, these attributes have additional relevance because they have been associated with the uniformity of drug distribution across the inserts [143,144,145,146]. Consistency in the physical characteristics of individual units has also been regarded as an indicator of the effectiveness and reproducibility of the manufacturing procedure [48,105], as well as a factor supporting the clinical applicability of the formulation [60,85].

#### 3.1.1. Thickness

Thickness has most commonly been assessed in anti-infective ocular insert formulations using Vernier, gauge or dead-weight callipers. Measurements were generally obtained from individual inserts, although uncut parent films were also examined [84]. For individual-unit assessment, between 3 and 24 specimens were selected, frequently at random [48,71]. Mean thickness was then calculated from repeated measurements taken at different positions on each specimen, with the number of readings ranging from 3 to 10 [74,83,147].

A comparable number of readings was used when uncut films were evaluated [83]. These measurements were obtained either from unspecified random locations or according to a predefined spatial pattern, such as the centre and four corners of the film [59]. Microscopic procedures have also been used to estimate mean thickness [57,105]. The corresponding specimen preparation and measurement plane were, however, not always described. Where sectioning or transverse cutting was not reported, the published values may reasonably be interpreted as representing the thickness at the film edge rather than through its central region [105].

#### 3.1.2. Weight

As with thickness, the assessment of individual insert weight may provide an indirect indication of formulation homogeneity and manufacturing repeatability, together with the consistency of API and excipient distribution across the film [48,105]. Mean formulation weight has most commonly been calculated from measurements obtained from three inserts, although larger numbers of specimens have also been examined [48,85,110,147].

The use of 20 units may provide a more robust basis for this assessment, as this sample size meets the requirement specified for pharmacopoeial uniformity-of-weight testing of single-dose solid preparations [139,148]. Such testing may be particularly informative for blank films, for which uniformity cannot be evaluated through drug-content analysis [143]. Nevertheless, the complete procedure has rarely been applied to anti-infective ocular insert formulations, including where a sufficient number of specimens was available for testing [48,98].

#### 3.1.3. Drug Content and Uniformity

Determination of drug content and verification of its uniformity among dosage units are fundamental requirements in the development of film-based drug-delivery systems [50]. In addition to confirming the quality of the manufactured product [140], these assessments are particularly relevant during formulation development because they provide evidence of manufacturing consistency and establish the drug quantity against which subsequent release measurements can be interpreted. Although closely related, drug-content assay and content-uniformity testing represent distinct evaluations with separate acceptance specifications. Drug-content assay determines the agreement between the nominal amount and the mean quantity measured across a batch, whereas content-uniformity testing evaluates variability among individual dosage units, initially using 10 randomly selected units [149].

Where an individual parent film was treated as the batch for sampling purposes and the inserts as its dosage units, the number of anti-infective ocular inserts assessed rarely met the initial threshold of 10 units for content-uniformity testing [48,57]. More commonly, between three and five specimens were examined [56,99,147], with comparable sample sizes used for determination of mean drug content [84,85,109,123]. Drug content and/or content uniformity were not consistently evaluated, and qualitative assessments of uniformity have also been reported [87]. Such qualitative observations may complement quantitative testing but should not be used as a standalone criterion.

The procedures used to recover and quantify the API may also provide information relevant to the manufacturing process. Poor content uniformity may, for example, indicate variability in the viscosity of the film-forming solution, which can hinder its complete transfer and uniform spreading over the casting surface [48]. For drug-content determination, inserts have been dissolved in defined volumes of different media, with phosphate-buffered saline and simulated tear fluid used predominantly for monolithic matrices [57,105,147], and organic solvents more commonly applied to multilayer inserts [83,128]. Crushing, agitation or sonication has also been used to promote dissolution of the insert and extraction of the incorporated API [84,85,98].

### 3.2. Physicochemical Properties of Inserts

Interactions between solid pharmaceutical dosage forms and environmental moisture require consideration throughout manufacture, storage and handling, as water uptake or loss may alter the characteristics of the finished product [150]. For solvent-cast films, this relationship is integral to manufacture, since film formation depends on the interactions among the solvent, API and excipients [151]. Following casting, the film-forming solution undergoes drying, and the conditions applied during this stage may influence both the residual solvent content and the properties of the resulting inserts [47]. Completion of drying may be inferred once film mass becomes stable over time, indicating approximate equilibrium between the formulation and the surrounding drying environment [110].

Even after drying, the film matrix may retain residual moisture and remain capable of exchanging water with the surrounding atmosphere. These complementary characteristics have commonly been evaluated in anti-infective ocular inserts through moisture-loss and moisture-absorption testing, conducted under low- and high-relative-humidity conditions, respectively.

#### 3.2.1. Moisture Loss

Moisture loss testing has been used to estimate the amount of moisture remaining after film drying and thereby support assessment of the post-drying condition of the inserts [42]. Residual moisture may depend on the affinity of the constituent polymers and plasticiser for the solvents used during manufacture and can influence film stickiness and mechanical behaviour [152,153]. Solvent retained within the matrix may also increase molecular mobility and act as an additional plasticiser, thereby contributing to insert flexibility [154,155].

For anti-infective ocular films, moisture loss has been evaluated by weighing specimens individually before storing them for three days in desiccators containing anhydrous calcium chloride at a relative humidity of 30 ± 5%. The result was expressed as the percentage reduction from the initial to the dried mass [99]. This procedure may nevertheless have limited capacity to determine the total quantity of solvent, particularly water, retained within the insert. Despite the limited thickness of film-based systems, continued solvent removal during drying may reduce the evaporation rate at the surface and leave moisture entrapped within the film matrix [156,157].

Several gravimetric procedures are available for assessing residual solvent or moisture in pharmaceutical formulations [158]. For buccal films, an oven-based loss-on-drying procedure has been widely applied, in which pre-weighed specimens were heated above 100 °C until a constant mass was obtained [48,136]. As exposure to elevated temperatures may alter polymeric components, the temperature and duration of the procedure can be adjusted to promote solvent removal from the film bulk while limiting thermal degradation [159].

#### 3.2.2. Moisture Absorption

Moisture absorption has generally been evaluated by weighing the specimens before storing them for three days in desiccators maintained at 75 ± 5% relative humidity using saturated solutions of aluminium chloride or, less commonly, sodium chloride. The result was expressed as the percentage change between the initial and final mass [83,110]. The extent of moisture uptake can nevertheless be strongly influenced by both relative humidity and temperature, with consequent changes in the molecular organisation and mechanical properties of the films [160].

Testing under these defined environmental conditions may, therefore, be more informative for establishing appropriate storage conditions than for predicting insert behaviour following ocular administration [161]. Measurement of water uptake from the surrounding atmosphere can support assessment of the physical stability of ocular films, but should primarily be interpreted as an indicator of hygroscopicity, as it does not represent the total amount of water that an insert may absorb after administration, given that the procedure does not reproduce direct contact with an aqueous medium or tear fluid [162,163].

#### 3.2.3. Water Uptake and Swelling Index

The pronounced capacity of film matrices to imbibe aqueous media may be attributed to the widespread use of hydrophilic excipients in these drug-delivery systems [47]. Penetration of water into the matrix can alter the organisation of the polymeric constituents, modifying the molecular structure of the insert and producing expansion or deformation of the device [142]. Assessment of water uptake may, therefore, assist in characterising how the insert interacts with the application site and how hydration contributes to drug release, potentially providing an indication of its behaviour after administration [142].

Water uptake in anti-infective ocular film inserts has been determined using different gravimetric procedures, generally by comparing the initial mass of the specimen with that measured after exposure to an aqueous medium. Simulated tear fluid has been used to provide closer approximation to the ocular environment, although phosphate buffer and distilled water have also been applied [48,57,105,129]. Specification of the soaking medium is essential for interpretation, as the extent of absorption can vary according to the solution used [164].

Monolithic matrices have most commonly been evaluated on agar gel plates, which supplied fluid to the inserts while the apparatus was maintained at a defined temperature, with specimens weighed at predetermined intervals until no further change in mass was observed [48,68,98]. For inserts that dissolve rapidly, undergo erosion or form a gel during hydration, the resulting change in mass requires cautious interpretation. Under these conditions, the measured value may reflect water uptake together with partial dissolution, matrix erosion, gel formation and loss of soluble components, limiting the ability of the procedure to distinguish swelling from progressive disintegration or other rapid physical transformations. Some procedures also incorporated pre-soaked filter paper and varied the incubation temperature, possibly facilitating specimen handling where substantial physical changes occurred during hydration [48,85,105]. Where preparation of the supporting substrate was insufficiently described, the amount of fluid available to the insert could not be established reliably [165]. Physical supports have also been used during the assessment of multilayer devices, while other procedures immersed the inserts directly in containers holding between 2 and 25 mL of the test medium, volumes that may not adequately represent ocular fluid availability [68,84].

While water uptake has been quantified gravimetrically, the swelling index has been defined from the relative increase in insert surface area or diameter following hydration, providing additional information on the potential retention characteristics of the device after administration. Swelling has been assessed by measuring changes in insert diameter at predetermined intervals after exposure to an aqueous medium until no further dimensional increase occurred [48,83], or by maintaining the inserts on ocular tissue models for specified periods [73,109]. The swelling index was subsequently expressed as the percentage ratio between the swollen and initial dry diameter, with image-analysis software also used to support quantitative measurement [73].

#### 3.2.4. Surface pH

Surface pH assessment may support identification of formulations with the potential to cause irritation or damage following contact with the ocular mucosa [145]. The measured value can be influenced by both the incorporated API and the identity and concentration of the other formulation constituents [166]. In anti-infective ocular film inserts, surface pH has been determined using either indicator paper or a digital pH meter [48,85,128].

As a dry film does not permit direct pH measurement, the insert must first be hydrated, and the conditions used for this step have varied considerably. Reported procedures ranged from applying 30 µL directly to the insert surface to immersing or exposing the specimen to as much as 5 mL of fluid [48,128]. Hydration periods were similarly heterogeneous, extending from approximately 30 s for directly soaked inserts to 5 h for specimens maintained on agar plates [98,123]. Distilled water was the most frequently selected medium, whereas simulated tear fluid was used less often [98]. These procedural differences limit direct comparison among reported values, as the measured pH reflects not only the formulation but also the volume, composition, and duration of contact with the hydrating medium.

The physiological relevance of the result also requires cautious interpretation. Despite its limited volume, the tear film possesses buffering capacity, while lacrimal turnover contributes to restoration of ocular-surface pH following exposure to exogenous formulations [167]. In the absence of specific guidance for ocular inserts, a more harmonised procedure could employ a reduced and clearly defined fluid volume, a standardised hydration period and a medium with controlled buffering properties. These conditions should be reported in full so that surface pH can be interpreted as a formulation-characterisation parameter rather than as an independent demonstration of ocular safety.

### 3.3. Mechanical Properties of Inserts

#### 3.3.1. Tensile Strength, Elongation and Young’s Modulus

Mechanical testing can provide information on the physical integrity and stability of film formulations [118]. No standardised procedure has been established specifically for determining these characteristics in ocular film inserts [152]. Consequently, different instruments and experimental procedures have been employed, frequently through adaptation of testing standards developed for other materials [136,147]. The principal parameters determined have been tensile strength, elongation at break and Young’s modulus. These properties are generally derived from the stress–strain curve (Figure 3), which illustrates representative relationships between the tensile stress applied to film specimens and the resulting strain, including examples of different mechanical behaviours [119]. Depending on the formulation, the curve may comprise an initial linear region corresponding to reversible elastic deformation, followed by a non-linear region associated with permanent plastic deformation and, ultimately, specimen rupture [168].

Tensile strength represents the maximum tensile stress sustained by the specimen during testing, whereas elongation at break describes the strain reached at rupture. Young’s modulus is calculated from the slope of the initial linear elastic region of the stress–strain curve and reflects the stiffness of the material [169]. Higher values of Young’s modulus are associated with greater rigidity, while lower values indicate a more flexible formulation [119,169]. Soft and weak inserts exhibit low tensile strength, Young’s modulus and elongation at break, whereas soft and strong films combine moderate tensile strength and low Young’s modulus with high elongation at break [118]. Although universal reference values cannot be applied, an ocular film insert should combine sufficient stiffness for handling with adequate flexibility in the dry state to permit adaptation to the ocular surface during application [152].

Mechanical properties of anti-infective ocular inserts have been examined using both computer-controlled testing instruments and purpose-built apparatuses capable of generating stress–strain data [48,87,98]. The procedural specifications were reported inconsistently and varied substantially among formulations. Specimens were prepared as rectangular strips, with reported dimensions ranging from 20 × 2 cm [147] to 50 × 10 mm [108], or as discs measuring 25 mm in diameter. Other reported parameters included pulling speeds ranging from 10 to 60 mm/min, an initial grip separation of 50 mm and a stress range of 0.05 MPa [48,147]. The limited and heterogeneous reporting of specimen geometry and testing conditions restricts direct comparison of mechanical-property values among formulations.

#### 3.3.2. Folding Endurance

Folding endurance provides an additional means of assessing the ability of film formulations to withstand repeated deformation, with potential relevance to insert fracture during storage, handling and administration [136]. The method was originally developed for paper, using purpose-built apparatuses to apply repeated folds under standardised conditions [170]. For anti-infective ocular film inserts, it has instead been used as a quantitative measure of flexibility or, more commonly, as an indicator of brittleness [68,94,108].

Reported procedures have predominantly relied on manual folding. Inserts were repeatedly folded along the same line by aligning and overlapping the specimen edges between two fingers [68,85,128,147], while forceps were used in another procedural variation [123]. Folding was continued until visible cracking or complete breakage occurred, and the number of repetitions completed at the selected endpoint was reported as the folding endurance value [84,98,105]. Interpretation of this value has not been entirely consistent. Although lower counts have sometimes been associated with adequate film flexibility, 300 repetitions has also been adopted as an empirical threshold for satisfactory performance [48,59,147].

Manual deformation may introduce operator-dependent variability, while the absence of a consistently defined endpoint can limit the accuracy and repeatability of the procedure. Greater standardisation could be achieved through a purpose-built apparatus that controls the folding motion and applies a predefined failure endpoint, consistent with the instrument-based principle on which folding-endurance testing was originally established [170]. Adaptation to polymeric ocular films would nevertheless require consideration of specimen geometry, conditioning and the intended interpretation of the resulting value.

### 3.4. Drug Release from Inserts

Characterisation of sustained-delivery formulations requires assessment of the amount of API liberated over time. Any in vitro procedure adopted or modified for this purpose should generate accurate, robust and reproducible data and should be capable of detecting meaningful changes in release behaviour arising from alterations to the formulation [31]. Experimental models that simplify the target tissue can make apparatus construction and operation more practicable, although this simplification may reduce their ability to reproduce the biological environment being represented [171]. Ocular drug-delivery systems have consequently been investigated using models designed around the anterior surface [172], anterior chamber [173] and posterior segment [31].

Methods reported for the in vitro evaluation of drug release from anti-infective ocular film inserts can be organised into three broad groups: vial-based procedures, flow-through systems and donor–receiver compartment models (Table 3). Vial-based testing offers the simplest arrangement. The insert is exposed to pre-heated dissolution medium, and aliquots withdrawn directly from the container are analysed to quantify release. The reported volume of medium has varied between 3 and 30 mL [59,129]. Movement intended to approximate blinking has been introduced by oscillating the vessel [83,84] or by stirring the medium. During stirred testing, the formulation has been enclosed within a dialysis-membrane bag or protected beneath a sieve [174]. A basket sieve has also been incorporated into an adaptation of a pharmacopoeial dissolution procedure originally intended for oral solid dosage forms, with a smaller quantity of medium used for ocular inserts [59].

Selection of the release medium introduced a further source of variability. Simulated tear fluid, also termed artificial tear fluid, did not denote a single composition, while compendial guidance did not prescribe a preparation specific to ocular-insert release testing [175]. The recurrent electrolyte basis contained sodium chloride (6.7–6.8 g/L), sodium bicarbonate (2.0–2.2 g/L) and calcium chloride dihydrate (0.08 g/L), with pH reported between 7.2 and 7.4 [56,98]. A closely related formulation contained calcium chloride (0.06 g/L), although its hydration state was not specified [109], while a potassium-containing variant additionally included potassium chloride (1.4 g/L) [48,176]. A compositionally distinct medium has also been reported, containing sodium chloride (0.658 g), calcium chloride (0.008 g), glucose (0.650 g), bovine serum albumin (0.268 g), globulin (0.134 g) and lysozyme (0.268 g) in water to 100 g at pH 7.2 [74,177]. Several investigations reported only the STF or ATF designation and pH. Since ionic and protein composition may influence matrix hydration, calcium-mediated gelation, erosion and API transport, the medium should be fully specified and interpreted alongside volume, temperature, renewal/flow and hydrodynamic conditions.

Although vial procedures are straightforward and may be useful for formulation screening, exposure to several millilitres of static or continuously mixed medium differs substantially from the restricted fluid volume, directional tear renewal and lacrimal drainage encountered following ocular administration [178,179,180]. Vessel oscillation and stirring also create bulk movement of the surrounding liquid rather than the repeated, localised interaction produced by blinking and movement of adjacent ocular tissues. These experimental conditions may alter the concentration gradient around the formulation and promote hydration, erosion or dissolution of the polymer matrix, thereby affecting both the extent and time course of the measured release.

Flow-through methods have generally used purpose-built apparatuses in which the insert is continuously irrigated within a dedicated chamber. One configuration maintained the formulation in a defined quantity of dissolution medium that was continually renewed [56]. In other systems, medium passed over the insert at rates ranging from 0.50 ± 0.10 mL/h to 10 drops per minute before collection in a receiving compartment for analysis [58,87]. A further arrangement positioned the specimen in a glass Petri dish inclined at 45° and supplied simulated tear fluid at 0.06 mL/min under room-temperature conditions of 19–22 °C [109]. The directional supply and collection of medium introduce features that are absent from a closed vial. The interpretation and comparability of these methods remain dependent on the initial fluid volume, chamber dimensions, insert orientation and support, distribution of liquid across the specimen, flow rate, residence time, temperature and evaporative loss. Flow expressed only as drops per minute is also difficult to compare unless the corresponding drop volume is defined. The quantity of medium supplied should, therefore, be considered alongside the quantity recovered, and potential retention of API within tubing, supports or other apparatus components should be taken into account when interpreting the release profile [181].

Donor–receiver systems use two separate chambers divided by a membrane, with dissolved API moving from the donor side containing the insert into the receptor phase. Franz diffusion cells constitute a specific form of this arrangement and permit different separating materials, including excised corneal tissue, while water-jacketed configurations provide control of temperature [57,182]. These systems have also been employed for in vitro or ex vivo evaluation of transcorneal permeability using sheep, rabbit and porcine corneas [57,85,105,110]. Where biological tissue is used as the separating barrier, the concentration measured in the receptor phase reflects API release from the insert together with subsequent permeation through the tissue and should not be considered representative of release alone. Regenerated-cellulose semi-permeable membranes, commonly used in the form of dialysis tubing, have represented the most frequent barrier in donor–receiver testing and have also been described as reproducing the corneal epithelial barrier [71]. Their primary role is, instead, to confine or separate the formulation while permitting passage of dissolved API into the receptor compartment. Regenerated cellulose should therefore not be considered equivalent to the corneal epithelium unless its barrier properties have been demonstrated to be suitable for that specific purpose. The volumes introduced into donor–receiver apparatuses have differed considerably. Between 3 and 60 mL of medium has been used in the receptor compartment [57,128], while reported donor quantities have extended from 0.7 µL to 1 mL [48,94,108]. The means by which the donor volume was kept constant was not always described, despite the water uptake propensity of the formulations and the liquid retained by membranes following pre-soaking. Where the donor quantity was omitted, it was also unclear whether the insert had been tested without addition of further medium [70]. The amount of fluid actually available for initial hydration, movement between compartments, evaporation and replacement after sampling should therefore be monitored and documented, rather than inferred solely from the nominal volume added at the beginning of the procedure.

Strong positive associations between in vitro and in vivo findings have been reported for donor–receiver systems using regenerated-cellulose membranes, with in vivo release generally inferred from the quantity of API remaining in the insert following placement within the rabbit conjunctival cul-de-sac [71,98,99]. Comparable associations were also described for flow-through apparatuses [56,58] and vial-based procedures [83]. Limitations in the ability of in vitro testing to reproduce the performance of topical ocular drug-delivery systems have nevertheless been identified for both film formulations [183] and medicated contact lenses [180]. The reported relationships may therefore be confined to specific apparatus-formulation combinations and provide limited evidence of reproducibility or transferability to other inserts. They are more appropriately interpreted as study-specific correspondence than as general evidence of in vitro–in vivo equivalence. Establishing procedures capable of predicting in vivo release remains pivotal to the advancement of sustained topical devices for ocular treatment [184].

Anterior-surface-mimicking apparatuses have progressively incorporated artificial tear delivery and blink-like motion and, although developed mainly for drug-eluting contact lenses, represent a methodological advance towards more dynamic and biorelevant testing [185,186]. Comparison of dynamic contact-lens models with tear-fluid measurements obtained in vivo has shown that correspondence may vary according to the API and experimental conditions, rather than demonstrating general equivalence between the apparatus and ocular performance [181]. More recent systems have additionally combined artificial lacrimal flow, an eyelid-like moving component and a cul-de-sac region relevant to formulations administered within the conjunctival sac [187,188]. Their suitability for thin film inserts and their capacity to predict insert behaviour in vivo remain unestablished. Until the fluidic and mechanical conditions can be reproduced consistently and their relationship with appropriate in vivo endpoints has been demonstrated, these systems should be regarded as mechanistic or comparative tools for formulation development rather than validated predictors of ocular performance [181,184].

As the available evidence does not support designation of a single apparatus as the reference method for all ocular film inserts, cross-study comparability may be strengthened through a minimum common testing and reporting framework applied irrespective of apparatus design. This framework should define the quantity and composition of fluid contacting the insert, temperature, specimen dimensions and orientation, agitation or flow conditions, sampling schedule, medium replacement, maintenance or absence of sink conditions, and the method used to calculate cumulative release [31,180,183]. Total API recovery, including drug remaining within the insert or retained by membranes and apparatus components, should also be considered, together with changes in matrix integrity during testing. A reproducible core procedure could thereby support formulation comparison and quality assessment, while dynamic or anatomically informed models may provide a complementary level of investigation where their added complexity is justified and progressively validated against in vivo performance.

### 3.5. Antimicrobial Efficacy

An anti-infective ocular insert should maintain antimicrobial activity as API release evolves over the intended exposure period. Direct testing of antimicrobial performance was nevertheless reported for only a minority of the formulations identified. Where undertaken, antimicrobial efficacy was most commonly assessed in vitro using agar-diffusion procedures and measurement of the resulting inhibition zone [48,57,70,123]. In these assays, the insert was placed on an inoculated agar surface and incubated under conditions supporting microbial growth; the resulting zone of inhibition was measured after microbiologically active API had been released from the formulation and diffused through the agar at concentrations sufficient to prevent visible growth [48,57,70,123]. Zone diameters were generally interpreted relative to a comparator, although results were also reported without a control formulation [70].

The selection of the comparator determines the interpretation that can be assigned to the measured response. Blank inserts were used to identify antimicrobial effects associated with the matrix constituents rather than the incorporated API [123,189], which is particularly relevant for polymers possessing intrinsic antimicrobial properties [190]. Marketed eye drops [98], ophthalmic ointments [83] and solutions containing an equivalent amount of API [87] were also employed, while some procedures incorporated both positive and negative controls on the same agar plate [57]. These comparators address different aspects of formulation performance: blank inserts indicate matrix-related activity, API solutions provide a response in the absence of a release-controlling matrix, and marketed formulations offer a reference against an established dosage form.

Some inserts produced inhibition zones larger than those generated by marketed formulations, although the basis of this finding was not established. In the absence of intrinsic antimicrobial activity from the matrix constituents, and provided that API dose and testing conditions are equivalent, an insert would not ordinarily be expected to generate a larger zone than the corresponding free-drug solution. Incorporation within a polymeric matrix should, instead, modulate hydration-dependent API liberation and its subsequent diffusion through the agar. Increasing polymer concentration has been associated with smaller inhibition zones, while differences in insert moistening altered antibiotic-release kinetics and, consequently, the magnitude of the measured response [57]. Individual polymers within blended matrices may also promote API release, indicating that the apparent antimicrobial effect can depend on the composition and relative proportions of the polymer system rather than on total polymer content alone [70]. Zone diameter should not, therefore, be interpreted directly as evidence of greater antimicrobial potency, and unexpectedly large inhibition zones should be interpreted with reference to the comparator formulation, API loading, possible matrix-related activity, hydration conditions and diffusion through the agar.

Time-dependent procedures have been used to extend antimicrobial evaluation beyond a single post-incubation endpoint. In one approach, ocular inserts were placed directly into *Staphylococcus aureus* suspensions prepared using different inoculum volumes, with microbial growth assessed at successive intervals by agar plating and a further microbial challenge introduced after 3 h [56]. In another, bacterial growth kinetics were monitored following exposure to antibiotic fractions collected at successive release time points. Preliminary findings indicated that the initial rapid-release phase produced an early antimicrobial response, while fractions obtained during the subsequent slower-release phase continued to inhibit growth throughout the remaining test period [191]. These approaches provide information on the persistence of antimicrobial activity but should be distinguished from conventional time-kill testing. Optical-density growth curves indicate growth inhibition and subsequent recovery, whereas time-kill assays quantify changes in viable-cell numbers through serial colony counts, thereby characterising the rate and extent of microbial killing and any later regrowth [192,193].

Antimicrobial performance was also inferred by comparing the concentrations of API released at selected time points with published minimum inhibitory concentration (MIC) values [73,108]. The microbial strains, susceptibility profiles and exposure conditions underlying the reported MICs were not always described sufficiently, limiting the relevance of these comparisons to the tested formulations. As an MIC represents the lowest concentration preventing visible growth of a specified microorganism under defined experimental conditions, exceeding a published value does not independently demonstrate microbial killing or confirm that inhibitory exposure is maintained throughout the intended release period [194,195].

Agar diffusion, broth-based testing, growth-kinetic monitoring and time-kill assays may therefore provide complementary rather than interchangeable evidence. Agar diffusion retains the intact insert and permits progressive hydration and API release, although the final inhibition zone provides limited information on changes in antimicrobial activity over time. Broth dilution offers a more standardised assessment of planktonic susceptibility and can be applied to the API itself or to fractions collected during a separately controlled release experiment [194]. Testing successive release fractions may indicate whether microbiologically active API remains available as release progresses, but does not evaluate hydration and API liberation from the intact insert within the microbiological assay [191]. Direct exposure of the insert to a microbial suspension instead combines antimicrobial assessment with formulation hydration and drug release under the selected broth conditions [56]. The comparatively large and uniformly mixed volumes commonly used in broth-based procedures may nevertheless alter matrix hydration and API delivery relative to the limited and continuously renewed fluid environment encountered at the ocular surface. Fluid volume, agitation and medium replacement should therefore be considered formulation-exposure variables rather than solely microbiological test conditions.

Time-kill procedures may add direct evidence of viable-cell reduction and subsequent regrowth. Biofilm assessment may also be considered where persistent, recurrent or device-associated infection is relevant, as biofilm formation on ocular devices can reduce antimicrobial susceptibility [196,197]. Contact-lens models provide a useful ocular-device precedent, although adaptation would be required to account for differences in insert composition, hydration behaviour, residence site and release profile [198,199].

The relevance of antimicrobial testing to the intended indication and anticipated exposure conditions should first be established, as not every assay will be equally informative for every formulation or target infection. Method selection should then reflect both the microbiological question being addressed and the way in which the insert is expected to release the API over time. Where appropriate, complementary procedures may be combined to assess the intact formulation, the activity of released drug and the persistence of the antimicrobial effect. Transparent and sufficiently detailed reporting of the experimental conditions would improve interpretation and comparison across formulations. As no single assay can reproduce both the evolving antimicrobial exposure and the ocular environment, the resulting in vitro findings should be considered supportive evidence of retained activity rather than direct demonstration of clinical efficacy.

## 4. Sterility, Ocular Biocompatibility and Tolerability

Sterility and ocular compatibility should be established for the finished insert rather than inferred from the suitability of individual constituents, physicochemical characterisation, or absence of visible irritation. Sterilisation was reported inconsistently for ocular anti-infective inserts, with limited consideration of process validation, packaging conditions, or post-treatment product performance [56,58,99,123]. Current good manufacturing practice favours terminal sterilisation where the formulation and packaging can withstand the selected process, while aseptic manufacture requires appropriate justification and process control where terminal treatment is unsuitable. Finished-product sterility testing represents only one component of sterility assurance and cannot compensate for an inadequately controlled or unvalidated manufacturing process [200]. Reported approaches included sterility testing, ultraviolet exposure followed by microbiological incubation, and gamma irradiation before biological evaluation [56,58,99,123]. Absence of microbial growth confirms the outcome of the applied test but does not establish that the manufacturing process consistently achieves sterility. This distinction is particularly relevant to ultraviolet exposure, which is not accepted as a sterilisation method under current EU good manufacturing practice [200]. Sterilisation compatibility is also formulation-specific, as heat, radiation, or chemical exposure may affect the API, polymeric matrix, residual moisture, mechanical behaviour, and drug-release profile. Process selection should therefore be supported by pre- and post-treatment evaluation of the critical chemical, physical, performance, biological, and packaging attributes of the finished insert.

Biological assessment was similarly fragmented. Dedicated cellular testing was reported for selected ciprofloxacin-loaded polymeric membranes and glycol-chitosan/oxidised-hyaluronic-acid films, while other formulations were assessed using Draize-type procedures or brief observations of redness, lacrimation, discharge, tissue appearance, or visible irritation [71,74,87,129]. Each endpoint addresses a distinct component of biological performance and should be interpreted accordingly. Cytocompatibility of the complete drug-loaded insert should be distinguished from that of the blank matrix and free API, while permeability measurements, surface pH, constituent-level biocompatibility, or gross tissue inspection cannot independently establish compatibility of the finished dosage form. Validated cellular and ex vivo ocular-irritation procedures can support a staged evaluation strategy, provided that exposure conditions account for the prolonged contact, progressive hydration, swelling, erosion, and potential repeated administration associated with an ocular insert [201].

Retention and ocular tolerability also require separate interpretation. Failure to expel an insert, prolonged residence, or apparent mucoadhesion describes dosage-form behaviour within the conjunctival sac but does not establish tissue compatibility or comfort [56,83,99]. In infected-eye models, redness, lacrimation, and discharge may additionally reflect the underlying condition, therapeutic response, device-related irritation, or a combination of these factors [58]. In vivo evaluation should therefore combine structured examination of corneal, iridal, and conjunctival responses with assessment of insert movement, expulsion, dissolution or erosion, fragmentation, and residual material over a formulation-relevant period. Formal patient-centred acceptability has not been characterised for the ocular anti-infective thin film inserts examined. Clinical progression will consequently require coordinated evidence on sterility assurance, post-sterilisation product comparability, ocular biocompatibility, in vivo tolerability and retention, and patient-centred outcomes.

## 5. Future Directions

Further advancement of ocular anti-infective thin film inserts should be organised around an indication-led and stage-appropriate development pathway, rather than continued optimisation of individual formulation attributes in isolation. The intended infection, relevant microbial spectrum, dose, treatment duration, administration site, and anticipated residence period should first be defined within a target product profile. Formulation design, manufacture, drug release, retained antimicrobial activity, ocular compatibility and practical administration could then be investigated as connected elements of the same development programme. Such integration is required because prolonged in vitro release, favourable mechanical properties or inhibition in an individual microbiological assay cannot independently establish clinically relevant performance [1,46,48,49].

The limited progression of historical anti-infective insert programmes provides an early illustration of the difficulty of sustaining this pathway beyond formulation feasibility. Soluble ophthalmic drug inserts containing antibacterial and antiviral agents were reported for clinical administration during the 1970s [36,202], while the patent associated with this platform specified preparation of the polymer–drug mixture, casting, controlled drying, residual-moisture limits, subdivision, sterilisation and packaging [203]. The later NODS platform incorporated controlled film manufacture and applicator-assisted placement, with several anti-infective agents included among the disclosed active substances [204]. These programmes demonstrate that progression to human administration was achievable, although the limited methodological and clinical detail available prevents appraisal against current evidential standards, and patent disclosures cannot substitute for independent evaluation of product quality or clinical performance. The limited continuity of these development programmes places renewed emphasis on reproducible manufacture and finished-product evaluation as prerequisites for subsequent clinical development.

Within this development pathway, the evidence available does not permit robust comparison of the industrial scalability of solvent casting, hot-melt extrusion, electrospinning and additive manufacturing. Future investigations should, therefore, progress beyond technical feasibility and define platform-specific material attributes, operating ranges, in-process controls and finished-product specifications. Reproducibility should be demonstrated across independently manufactured batches and should encompass dosage-unit uniformity, structural integrity, drug stability, release behaviour and antimicrobial performance. Scale-up should additionally account for equipment configuration, continuous or batch processing, subdivision into dosage units, post-processing, packaging and storage, rather than being treated as a direct enlargement of laboratory manufacture [47,52,55,62].

Additive manufacturing may provide digitally controlled modification of insert geometry, internal structure and drug content, with potential relevance to more individualised products. The current evidence demonstrates technical feasibility more clearly than a clinical advantage over standardised dosage units or readiness for routine manufacture. Future research should establish whether product customisation produces a meaningful therapeutic or practical benefit and whether the resulting units can be manufactured reproducibly within an appropriate pharmaceutical quality system. Comparative designs should also incorporate resource use, processing time, equipment and personnel requirements, post-processing, manufacturing failures and quality-control burden. Without such evidence, no manufacturing route can presently be identified as intrinsically the least expensive or most reliable, and pharmacoeconomic advantage should not be inferred from procedural simplicity or laboratory-scale production costs alone [54,67,70].

Sterility assurance, ocular compatibility, and regulatory quality should be incorporated prospectively into formulation, manufacturing, and packaging development. Comparative evidence remains insufficient to support a sterilisation method that can be expected to preserve all APIs, excipients, and film architectures. Each product should therefore be linked to a justified terminal-sterilisation or aseptic-manufacturing strategy, supported by predefined assessment of the critical chemical, physical, performance, biological, and packaging attributes following processing. Established requirements for ophthalmic preparations and single-dose products may provide the regulatory foundation, while insert-specific criteria for hydration, matrix transformation, residence, and antimicrobial performance will require coordinated evidence before broader standardisation can be proposed [46,136,138,139,205].

Evaluation should subsequently progress through staged and increasingly biorelevant investigation. Drug-release and antimicrobial methodologies should account more explicitly for the limited and continuously renewed fluid volume encountered after conjunctival administration, together with lacrimal inflow, drainage, insert residence and time-dependent drug availability. The microbial model and efficacy endpoint should be selected in relation to the intended indication and anticipated exposure, with complementary methods used where necessary to distinguish API release from retained microbiological activity. Later-stage studies should additionally examine insertion, positioning, retention, dissolution or removal, comfort, visual interference and user acceptability alongside ocular safety and therapeutic performance. This pathway would allow clinical evaluation to examine a sufficiently defined product, rather than relying on clinical investigation to resolve persistent variability in formulation, manufacture or in vitro assessment [1,25,46,49].

## 6. Conclusions

Ocular anti-infective thin film inserts provide a means of administering a defined drug-containing unit within the conjunctival sac, with the potential to extend local residence and reduce dependence on frequent eye-drop instillation. Their relevance as an ocular dosage form depends on whether the finished insert can be manufactured consistently and behave predictably after administration while maintaining active drug availability and ocular compatibility.

Formulation design and manufacture are closely interdependent. Material characteristics shape processability and film formation, while processing conditions may influence the uniformity and subsequent behaviour attributed to the formulation. Solvent casting remains the most extensively applied approach and has established the feasibility of a broad range of film designs, although incomplete specification of materials and manufacturing conditions frequently limits reproducibility and comparison. Alternative technologies extend the available manufacturing possibilities, with their suitability remaining dependent on the intended characteristics and use of the resulting product.

Comparable limitations affect the interpretation of testing, as heterogeneous procedures often examine isolated attributes without establishing how the finished insert performs under conditions relevant to ocular administration. The absence of insert-specific compendial or regulatory standards further increases the importance of appropriately justified methods and sufficiently complete reporting. Progression beyond formulation feasibility will therefore depend on coherent product development in which manufacturing control and evaluation are aligned with the intended clinical application. This approach would provide a stronger basis for determining whether the performance observed under laboratory conditions can translate into a reproducible, safe and clinically relevant ocular dosage form.

## Figures and Tables

**Table 1:** Main characteristics, potential advantages and principal limitations of manufacturing strategies investigated for ocular anti-infective inserts. [API, active pharmaceutical ingredient; FDM, fused-deposition modelling; HME, hot-melt extrusion].

Main Characteristics	Principal Advantages	Potential Limitations
**Solvent casting**		
API dissolved, dispersed or incorporated through a carrier into a polymer-containing liquid, cast directly within unit-defining moulds or deposited onto a larger substrate and dried to form a parent film for subsequent subdivision [48,56,57]. Sequential casting or lamination can generate bilayer and reservoir systems [58,59,60]. Control centres on precursor homogeneity and rheology, air removal, deposition, drying and subdivision [48,57].	Accommodates dissolved APIs and carrier-loaded systems in aqueous or mixed-solvent formulations [48,61]. Monolithic and multilayer architectures can be produced without melt processing [48,60].	Interdependent formulation, casting and drying variables may compromise spatial and batch consistency [48,62]. Organic-solvent processing requires solvent-specific removal and residual assessment, while multilayers add interfacial-control requirements [59,60].
**Hot-melt extrusion**		
Drug-excipient blends are thermo-mechanically mixed and extruded for direct subdivision [52,63] or as FDM filament [54]. Control centres on feed consistency, softening and plasticisation, thermal-shear exposure, residence time and extrudate geometry [52,63]. Filament manufacture additionally requires dimensional consistency and mechanical feedability [54].	Avoids processing solvents and combines mixing with shaping [52,63]. The extrudate can also provide printable feedstock within an integrated HME-FDM process [54].	Application is constrained by API and excipient thermomechanical stability and reliable extrudate formation [52,63]. Evidence remains confined to laboratory-scale extrusion, with limited assessment of scale-up and independent-run reproducibility [52,63].
**Electrospinning**		
Electrically driven polymer jets form non-woven fibrous matrices [64,65]. Sequential deposition or crosslinking can generate multilayer or stabilised systems [53,64,66]. Control centres on solution properties, electrical field, delivery, working distance, environmental conditions, deposition and post-treatment [64,65,66].	Sequential deposition can separate drug-containing and barrier functions between fibrous layers [53,66]. Crosslinking or hydrophobic outer layers can reduce hydration and slow drug release [53,64].	Incomplete reporting of environmental and deposition conditions restricts reproducibility [53,66]. Dose definition requires controlled deposition and subdivision, while crosslinking introduces residual-reactant and biocompatibility concerns [64,65].
**Additive manufacturing**		
Reported routes comprise HME-fed FDM [54], semi-solid extrusion [67] and direct powder extrusion [68]. Critical elements are platform-specific: filament feedability for FDM [54], rheology and shape retention for semi-solid extrusion [67], and powder transport, melting and strand formation for direct powder extrusion [68].	Digital control permits deliberate variation of thickness and infill [54,68] or deposited layer number [67]. Distinct platforms accommodate filament, semi-solid and powder feedstocks [54,67,68].	Thermal exposure, rheological requirements, resolution and post-processing cannot be generalised across platforms [54,67,68]. Actual-to-design accuracy, failed-print yield and reproducibility across independent runs remain incompletely characterised [54,67,68].

**Table 2:** Key attributes, formulation roles and selection considerations of polymers and plasticisers investigated for ocular anti-infective thin film inserts. [API, active pharmaceutical ingredient; CAP, cellulose acetate phthalate; CMC, carboxymethyl cellulose; EC, ethyl cellulose; FDA-IID, United States Food and Drug Administration Inactive Ingredient Database; HPMC, hydroxypropyl methylcellulose; PEG, polyethylene glycol; PVA, polyvinyl alcohol; PVP, polyvinylpyrrolidone; t90, time required for 90% cumulative drug release].

Key Attributes and Formulation Role	Aqueous Behaviour and Formulation Effects	Critical Formulation Variables and Limitations
**Hydroxypropyl methylcellulose (HPMC)**—semi-synthetic non-ionic cellulose ether [82]		
Hydrophilic film former; drug-containing matrix or layer in monolithic, bilayered and reservoir systems [60,83,84,85]. Mucoadhesive contribution in a bilayered film [60].	Hydrated gel-layer formation; grade-, concentration- and composition-dependent swelling and erosion [60,82]. Non-ionic, without pH-triggered ionisation [82]. Diffusion- and erosion-mediated drug transport [60,80].	Substitution pattern and viscosity grade influence hydration and gel formation; concentration and blend ratio additionally affect precursor viscosity and drug transport [60,82]. Precursor viscosity, layer thickness and barrier composition [83,84,85]. Rapid erosion may shorten release; greater matrix density or external barriers may restrict transport excessively [60,83,84].
**Carboxymethyl cellulose (CMC)**—semi-synthetic anionic cellulose ether [86]		
Viscosity-enhancing, adhesive blend component [86,87]. Hydration-modifying phase, with PVA providing mechanical support [87].	Ready wetting and swelling; increased aqueous viscosity [86,87]. PVA-containing blend: sustained mucoadhesion, matrix degradation and antibiotic release (4–5 days) [87].	Grade, concentration and degree of substitution [86]. PVA:CMC ratio and total polymer content influence hydration, film strength and release; CMC-specific effects were not isolated from PVA [87]. Adhesion, degradation and release contributions not isolated from PVA [87].
**Ethyl cellulose (EC)**—semi-synthetic non-ionic cellulose ether with limited aqueous solubility [88]		
Film-forming external membrane for rate control around hydrophilic reservoirs [56,84].	Restricted water ingress and drug transport [56,88]. Slowest release among membranes surrounding a common PVA reservoir (85.80% at 24 h; RS100 93.85% at 24 h; RL100 98.71% at 21 h) [56].	Grade, membrane concentration, thickness, continuity and plasticisation [56,84]. Poor aqueous solubility: total drug recovery and post-release membrane fate require definition [46,49].
**Cellulose acetate phthalate (CAP)**—semi-synthetic ionisable cellulose ester [89]		
pH-responsive external membrane for rate control around hydrophilic reservoirs [83].	Increasing ionisation and dissolution above the grade-specific pH threshold [89]. Greater release control at 5% membrane concentration than at lower tested concentrations [83].	Polymer grade and dissolution threshold; membrane concentration and thickness [83,89]. Performance dependent on reservoir composition and architecture [83]. Test-medium pH requires control because ionisation, membrane dissolution and release are pH-dependent [89].
**Alginate and modified alginates**—natural anionic polysaccharides [90,91]		
Mucoadhesive, gel-forming matrix; monolithic, blended and reservoir designs [72,73,75]. Thiolation for increased ocular-surface retention [59].	Calcium-responsive gelation, including interaction with tear-fluid ions [91]. Drug transport through the swollen matrix with superficial gel-layer erosion [73]. Following CaCl_2_ treatment, longer t90 with crystalline API than soluble salt (23.8–25.8 vs. 8.9–16.6 h); further prolongation with HPMC (8.9 to 16.6 h; 23.8 to 25.8 h) [73].	Mannuronic:guluronic-acid composition, molecular grade, concentration and blend ratio [90,91]. CaCl_2_ concentration and exposure (4% *w*/*v* for 15 s selected; 60 s reduced flexibility), API physical state and HPMC incorporation may influence release alongside crosslinking [73]. Derivatisation and coating may modify swelling, accessible mucoadhesive groups and transport [59,75].
**Chitosan and derivatives**—natural cationic polysaccharides [92]		
Mucoadhesive film component; permeation-modifying and intrinsic antimicrobial potential [93,94,95]. Lysozyme-mediated biodegradability [92,96].	Acidic-media solubility following protonation; reduced native-polymer solubility near physiological pH [92,95]. Charge density, hydration and mucin interaction vary with molecular weight, deacetylation and derivatisation [92,97].	Molecular weight, degree of deacetylation, source, derivatisation and formulation pH [92,95]. Degree of deacetylation only occasionally reported in identified insert formulations [94,98,99]. Intrinsic antimicrobial activity may confound API-specific testing [93]. Not represented among excipients in approved human ophthalmic products [100].
**Gelatin**—natural protein-derived polymer [101,102]		
Biocompatible and biodegradable film-forming, gelling and adhesive matrix component [94,101,102]. Commonly blended (e.g., PVA or chitosan) for mechanical support [74,94].	Hydration and gel formation; concentration- and crosslinking-dependent persistence [71,74]. At 60 min hardening, higher concentration associated with longer near-complete release (16%: 98.21% at 8 h; 18%: 96.13% at 10 h) [71].	Biological source, gelatin type, molecular-weight distribution and concentration may affect gel formation, hydration and mechanical properties [102]. Limited mechanical strength without blending or modification [74]. Concentration and hardening duration influenced release; the absence of an uncrosslinked comparator limits attribution specifically to crosslinking [71]. Glutaraldehyde concentration and exposure, followed by sodium-metabisulphite treatment and washing, require control to minimise variability and residual crosslinker [71].
**Gellan gum and guar gum**—natural polysaccharide gums [103,104]		
**Gellan:** film-forming, ion-responsive gel matrix; standalone or blended use [99,104]. **Guar:** high-viscosity hydrophilic matrix and film former [103,105].	**Gellan:** ion-mediated gelation and 24 h sustained release; faster release at the higher PEG 400-to-polymer ratio [99,104]. **Guar:** increasing concentration increased swelling and tensile characteristics while reducing 24 h release (75.21% to 38.19%) [105].	**Gellan:** concentration and ionic composition influence gelation and precursor viscosity; PEG 400-to-polymer ratio affects drug release, while blend composition determines film-forming and processing behaviour [99,104]. **Guar:** molecular weight and concentration govern precursor viscosity; increasing concentration improves swelling and tensile characteristics but may hinder casting and restrict drug transport [103,105].
**Polyvinyl alcohol and polyvinylpyrrolidone (PVA/PVP)**—synthetic hydrophilic polymers [106,107]		
**PVA:** film-forming and mechanical backbone in monolithic matrices and blends; hydrophilic reservoir in membrane-controlled systems [56,74,87]. **PVP:** film-forming, adhesive and solubilising component used with hydrophilic matrices and poorly soluble barrier polymers [56,107,108].	**PVA:** rapid release from plain matrices (approximately 5 h); moderation of burst release by CMC or gelatin and extension beyond 20 h with external membranes [56,74,75,87]. Lower hydrolysis degree was associated with slower release in hyaluronic-acid-containing films [109]. **PVP:** blend composition altered viscosity and release duration; no clear sustained-release advantage over PVA alone [108]. Greater corneal penetration in one multicomponent soluble insert; polymer-specific contribution not isolated [110].	**PVA:** molecular weight and degree of hydrolysis influence hydration and swelling; concentration, total solids and blend ratio affect castability, mechanics and release [106,109]. Increasing PVA/Soluplus^®^ content slowed release, but further increases produced excessive viscosity and impaired casting [57]. External barriers required for prolonged release [56,75]. **PVP:** molecular weight or viscosity grade, concentration, PVA:PVP ratio and total solids affect precursor viscosity and blend behaviour; polymer-specific release effects remain difficult to isolate [107,108]. Observed effects dependent on barrier grade, accompanying excipients, and API characteristics [108,110].
**Polymethacrylates (Eudragit^®^ grades)**—permeability-controlled and pH-responsive synthetic copolymers [111,112]		
**RL/RS:** poorly water-soluble, permeable rate-controlling membranes or coatings [56,58,72,75]. **E/L/S:** ionisable matrices, coatings or membranes for pH-dependent release modification [75,108]. **L100:** nanoparticle carrier incorporated within a PVA film [61].	**RL/RS:** greater permeability of RL than RS; increasing RL:RS ratio accelerated release from an alginate reservoir [58,111,112]. Membrane release-rate order around a common PVA reservoir: RL100 > RS100 > EC (98.71% at 21 h; 93.85% at 24 h; 85.80% at 24 h); RS100 maintained in vivo release for 24 h [56]. In coated PVA–alginate inserts, RL100 prolonged release most (9 h), followed by RS100 (7 h) [75]. **E/L/S:** acidic-media dissolution for E and grade-specific pH thresholds for L/S [111,112]. Relative to the uncoated control (5 h), S100 extended release (7 h), E100 added no prolongation beyond crosslinking (6 h), and L100 remained constant (5 h) [75].	**RL/RS:** exact grade and ratio; membrane concentration, thickness, continuity, plasticisation, coating procedure and underlying matrix [56,58,72,75]. **E/L/S:** exact grade, medium pH and ionic composition; coating quantity and uniformity, matrix composition and API distribution [75,108,111,112]. Limited transferability across grades, ratios and architectures [75,108].
**Plasticisers**, principally PEG and glycerol; occasional use of dibutyl phthalate [75,84,113]		
Reduced intermolecular association; increased chain mobility, flexibility and handling [60,71,78,113]. PEG 400 and glycerol commonly used in hydrophilic and bilayered systems [60,71,94,99].	Concurrent changes in hydration, moisture uptake, thickness, permeability and release [60,78]. Higher PEG 400-to-polymer ratios accelerated release from gellan-, chitosan- and gelatin-containing films [94,99].	Plasticiser identity, molecular grade, concentration, distribution between layers and plasticiser-to-polymer ratio jointly affect flexibility, hydration and drug release [60,78,94,99]. Polymer compatibility and migration; mechanical and release effects require joint assessment [78,114]. Molecular grade unspecified in some formulations; higher PEG molecular weight associated with lower tensile strength and greater elongation in non-ocular films [58,115]. Limited relevance of dibutyl phthalate to new ocular formulations owing to toxicological concerns [75,84,116].

**Table 3:** In vitro, ex vivo and in vivo drug-release and associated evaluation methods reported for ocular anti-infective inserts. STF (simulated tear fluid), PBS (phosphate buffered saline).

Evaluation	In Vitro/Ex Vivo Drug-Release Apparatus and Method	Key Specifications	Release Medium	Medium Volume (Receiver/ Donor)	Time Tested	Associated In Vivo or Ex Vivo Evaluation	Reference
In vitro + in vivo	Static vial immersion	Static conditions	Sørensen buffer	20 mL	Up to 57 h	Tear-fluid drug analysis in rabbits	[73]
In vitro + in vivo	Vial immersion	Oscillating water bath at 32 °C	PBS	10 mL	20 h	Residual drug content in inserts and drug concentration in aqueous humour in rabbits	[83]
In vitro + in vivo	Vial immersion	Oscillating water bath at 32 °C	PBS	10 mL in a 15 mL vial	20 h	Residual drug content in inserts recovered from rabbit eyes	[84]
In vitro	Modified USP Apparatus I	Basket rotating at 50 rpm	STF	30 mL	Up to 12 h	Not performed	[59]
In vitro	Static immersion in a Petri dish	Insert immersed in a PBS-filled Petri dish	PBS, pH 7.4	20 mL	6 h	Not performed	[60]
In vitro + ex vivo + in vivo	Vial immersion with sieve	Stirred at 34 °C	PBS containing 5% RMβCD	10 mL	2–3 h	Evaluation using enucleated eyes and live rabbits	[174]
In vitro + in vivo	Static vial immersion	Temperature-controlled air bath at 37 °C	PBS	3 mL	1 h (in vitro); 24 h (tolerance)	Ocular tolerance in rabbits	[129]
In vitro + in vivo	Continuous flow-through system	Flow rate of 10 drops/min	STF	250 mL/50 mL	120 h	Residual drug content in inserts and drug concentration in aqueous humour in rabbits	[58]
In vitro + in vivo	Open flow-through cell	No externally driven flow; insert supported on a PTFE disc	STF	2 mL	24 h	Residual drug content in inserts recovered from rabbit eyes	[56]
In vitro + ex vivo	Continuous flow-through system	Flow rate of 0.50 ± 0.10 mL/h; fluorescein tracer	PBS	Flow-controlled; fixed volume not reported	Up to 48 h (in vitro); 12 h (ex vivo)	Evaluation using goat eyes	[87]
In vitro + ex vivo	Continuous flow-through system	Flow rate of 0.60 ± 0.15 mL/h; fluorescein tracer	PBS	Flow-controlled; fixed volume not reported	24 h (in vitro); 3 h (ex vivo)	Evaluation using goat eyes	[74]
In vitro + in vivo	Donor–receiver system	Pre-soaked commercial semipermeable cellophane membrane	STF	25 mL/not reported	Up to 12 h	Residual drug content in inserts recovered from the rabbit cul-de-sac	[98]
In vitro + in vivo	Donor–receiver system	Regenerated-cellulose dialysis membrane used as a corneal-barrier surrogate	PBS	25 mL/7 µL	10 h	Residual drug content in inserts recovered from the rabbit cul-de-sac	[71]
In vitro + in vivo	Donor–receiver system	Regenerated-cellulose dialysis membrane	PBS	25 mL/0.7 µL	24 h	Residual drug content in inserts recovered from the rabbit cul-de-sac	[99]
In vitro + in vivo	Donor–receiver system	Regenerated-cellulose dialysis membrane	PBS	Not reported/0.7 µL	24 h	Residual drug content in inserts recovered from the rabbit cul-de-sac	[94]
In vitro + in vivo	Donor–receiver system	Dialysis membrane; agitation at 20 rpm to simulate blinking	PBS	60 mL/5 µL	6 h	Residual drug content in inserts recovered from rabbit eyes	[128]
In vitro	Donor–receiver system	Dialysis membrane; stirred at 100 rpm and 37 °C; evaporation prevented	PBS	49 mL/1 mL	24 h	Not performed	[108]
In vitro + in vivo	Donor–receiver system	Cylindrical glass tube; shaking water bath at 37 ± 0.5 °C	PBS	25 mL/not reported	24 h (in vitro); 8 h (in vivo)	Residual drug content in inserts and drug concentration in aqueous humour in rabbits	[123]
Ex vivo	Modified Franz diffusion cell	Sheep cornea	STF	11 mL/not reported	24 h	Not performed	[105]
In vitro + ex vivo + in vivo	Franz diffusion cell	Fresh sheep cornea	STF, pH 7.4	11 mL/not reported	24 h	Ocular tolerance in rabbits	[85]
In vitro + ex vivo	Franz diffusion cell	Rabbit cornea under sink conditions	PBS	4.2 mL/not reported	3 h	Not performed	[110]
In vitro + ex vivo	Franz diffusion cell	Porous membrane (in vitro); porcine cornea (ex vivo)	PBS	3 mL/150 µL	24 h (in vitro); 6 h (ex vivo)	Not performed	[57]

PBS (phosphate buffered saline); STF (simulated tear fluid); RMβCD (Randomly Methylated Beta-Cyclodextrin).

## Data Availability

No new data were created or analysed in this study. Data sharing is not applicable to this article.
